# DNA Origami‐Based CD44‐Targeted Therapy Silences Stat3 Enhances Cartilage Regeneration and Alleviates Osteoarthritis Progression

**DOI:** 10.1002/advs.202503939

**Published:** 2025-05-21

**Authors:** Qi Lv, Xiang Zhao, Songsong Teng, Xinmeng Jin, Ying Zhou, Yueyang Sun, Hao Pei, Zuoqin Yan, Chunhui Ma

**Affiliations:** ^1^ Department of Medical Imaging Tongji Hospital School of Medicine Tongji University Shanghai 200065 China; ^2^ Department of Surgery of Spine and Spinal Cord Henan Provincial People's Hospital Zhengzhou 450003 China; ^3^ School of Chemistry and Life Sciences Suzhou University of Science and Technology Suzhou 215009 China; ^4^ Joint Laboratory of Biomaterials and Translational Medicine Puheng Technology Co., Ltd Suzhou 215000 China; ^5^ Department of Orthopedic Surgery Shanghai General Hospital Shanghai Jiao Tong University Shanghai 200080 China; ^6^ Shanghai Key laboratory of green chemistry and chemical Processes School of chemistry and Molecular engineering Shanghai center of Brain inspired intelligent Materials and devices East China normal University Shanghai 200241 China; ^7^ Department of Orthopedic Surgery Shanghai Geriatric Medical Center Shanghai 201104 China; ^8^ Institute of Bone and Joint Diseases Zhongshan Hospital Fudan University Shanghai 200433 China

**Keywords:** CD44 targeting, DNA triangle origami, osteoarthritis, si‐Stat3

## Abstract

Osteoarthritis (OA) is a widespread musculoskeletal disorder affecting ≈600 million people globally, and small interfering RNA (siRNA) therapy shows potential in targeting OA progression. However, the efficient and targeted delivery of siRNA remains a major challenge due to issues with tissue specificity and degradation in vivo. In this study, A DNA origami‐based chondrocyte‐targeted delivery system (OCS) is designed for siRNA delivery to OA‐affected cartilage. The DNA origami is engineered to load with siRNA targeting signal transducer and activator of transcription 3 (Stat3), a key regulator of inflammation and cartilage degradation, and is functionalized with anti‐CD44 aptamers for selective targeting of OA chondrocytes. In vitro, the DNA origami system effectively delivers siRNA to diseased chondrocytes, silencing matrix metalloproteinases expression and reducing inflammation. In OA rat models, it preserves cartilage integrity, promotes regeneration, and mitigates ECM degradation without evident side effects. These findings highlight DNA origami as a promising platform for siRNA‐based OA therapy, offering a promising solution to the challenges of targeted and efficient siRNA delivery.

## Graphical abstract

This study develops a cartilage‐targeted DNA origami system (OCS) that protects and delivers si‐Stat3 to chondrocytes. OCS reduces MMP13 expression, enhances extracellular matrix stability, and lowers oxidative stress while improving cell survival. In vivo, OCS decreases cartilage degradation biomarkers and promotes tissue repair factors, demonstrating a precision therapeutic strategy for osteoarthritis.

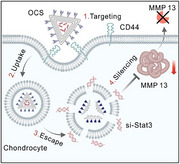

## 1 Introduction

Osteoarthritis (OA) is a prevalent degenerative joint disease affecting over 600 million people worldwide.^[^ ^1^, ^2^ ^]^ As global rates of aging, obesity, and joint injuries rise, the medical burden of OA is expected to grow significantly.^[^ ^3^, ^4^, ^5^ ^]^ Despite its high prevalence and severity, current clinical guidelines primarily recommend nonpharmacological interventions (e.g., exercise, weight management) and palliative pharmacological agents (e.g., NSAIDs, paracetamol) for symptom relief, with no therapies proven to restore lost cartilage or halt disease progression.^[^ ^6^ ^]^ Although OA is a multifactorial disease affecting articular cartilage, subchondral bone, and synovium, articular cartilage degeneration is a hallmark event in its pathology.^[^ ^7^, ^8^, ^9^, ^10^ ^]^ Therefore, it is important to develop new strategies to intervene in the homeostasis of articular cartilage at the onset and promote cartilage regeneration throughout the progression of OA.

Articular cartilage consists of chondrocytes and extracellular matrix (ECM). Chondrocytes maintain ECM homeostasis but shift to a catabolic phenotype under pathological stimuli such as mechanical stress or inflammation. This leads to the secretion of proinflammatory cytokines (e.g., IL‐1β, IL‐6, TNF‐α), which activate the Janus kinase 2/signal transducer and activator of transcription 3 (Jak2/Stat3) pathway.^[^ ^11^, ^12^, ^13^ ^]^ Activated Stat3 promotes the expression of matrix metalloproteinase 13 (MMP13), which degrades type II collagen (COL‐II), the primary structural protein in cartilage ECM.^[^ ^14^, ^15^ ^]^ Increased MMP13 activity accelerates ECM breakdown, contributing to OA progression. Therefore, targeting Stat3 to downregulate MMP13 offers a promising strategy to preserve cartilage integrity.^[^ ^16^, ^17^ ^]^ Silencing Stat3 with small interfering RNA (siRNA) has shown potential in reducing inflammation and cartilage degradation.^[^ ^18^, ^19^, ^20^ ^]^ However, siRNA is vulnerable to enzymatic degradation and has a short half‐life in circulation, limiting its therapeutic potential. Conventional delivery systems, such as liposomes, polymers, dendrimers, and viral vectors, face limitations related to stability, immunogenicity, and targeting specificity.^[^ ^21^, ^22^, ^23^, ^24^ ^]^ While liposomes can protect siRNA from degradation and facilitate cellular uptake, they may trigger immune responses^[^ ^25^, ^26^ ^]^ and viral vectors pose long‐term safety concerns.^[^ ^27^ ^]^ Additionally, conjugating siRNA with antibodies, aptamers, or peptides to target specific cells has proven challenging in achieving precise modifications and controlled targeting.^[^ ^28^, ^29^, ^30^ ^]^

DNA origami has recently emerged as a highly programmable and precise nanoplatform for therapeutic delivery. By utilizing specific DNA sequences, it can self‐assemble into highly precise, customizable nanostructures capable of carrying therapeutic agents like siRNA for targeted delivery.^[^ ^31^, ^32^, ^33^ ^]^ Its polyhedral architecture not only provides a protective barrier for siRNA, shielding it from enzymatic degradation, but also facilitates multivalent binding, enhancing interactions with target cells, promoting cellular uptake, and improving therapeutic efficacy.^[^ ^34^, ^35^, ^36^, ^37^ ^]^ Its potential in treating inflammatory diseases has also been demonstrated. For example, Ding et al.^[^ ^38^ ^]^ developed DNA nanoparticles that co‐deliver siRNA and anti‐inflammatory agents for rheumatoid arthritis, effectively modulating joint inflammation. Furthermore, functionalizing DNA origami with targeting ligands allows for selective tissue accumulation and reduced off‐target effects.^[^ ^39^, ^40^ ^]^

Here, we presented a novel DNA origami‐based chondrocyte‐targeted delivery system (OCS) for OA therapy. The DNA origami was designed to load with siRNA targeting si‐Stat3 at its core, providing protection from nuclease degradation and enhancing endosomal escape. To enhance targeting specificity, anti‐CD44 DNA aptamers were conjugated to the DNA origami, enabling selective binding to CD44 receptors, which are overexpressed on diseased chondrocytes.^[^ ^41^, ^42^ ^]^ This strategy greatly enhanced siRNA enrichment in OA‐affected chondrocytes, efficiently silencing the MMP13 expression and reducing inflammation. In OA cellular models, the system decreased ROS production, promoted chondrocyte viability, and inhibited inflammation. In OA rat models, it decreased cartilage degradation biomarkers, increased the expression of anabolic factors, and prevented ECM degradation while promoting cartilage regeneration, without noticeable side effects.

## 2 Results and Discussion

### 2.1 Construction of the DNA Origami‐Based Chondrocyte‐Targeted Delivery System

**Figure 1:**
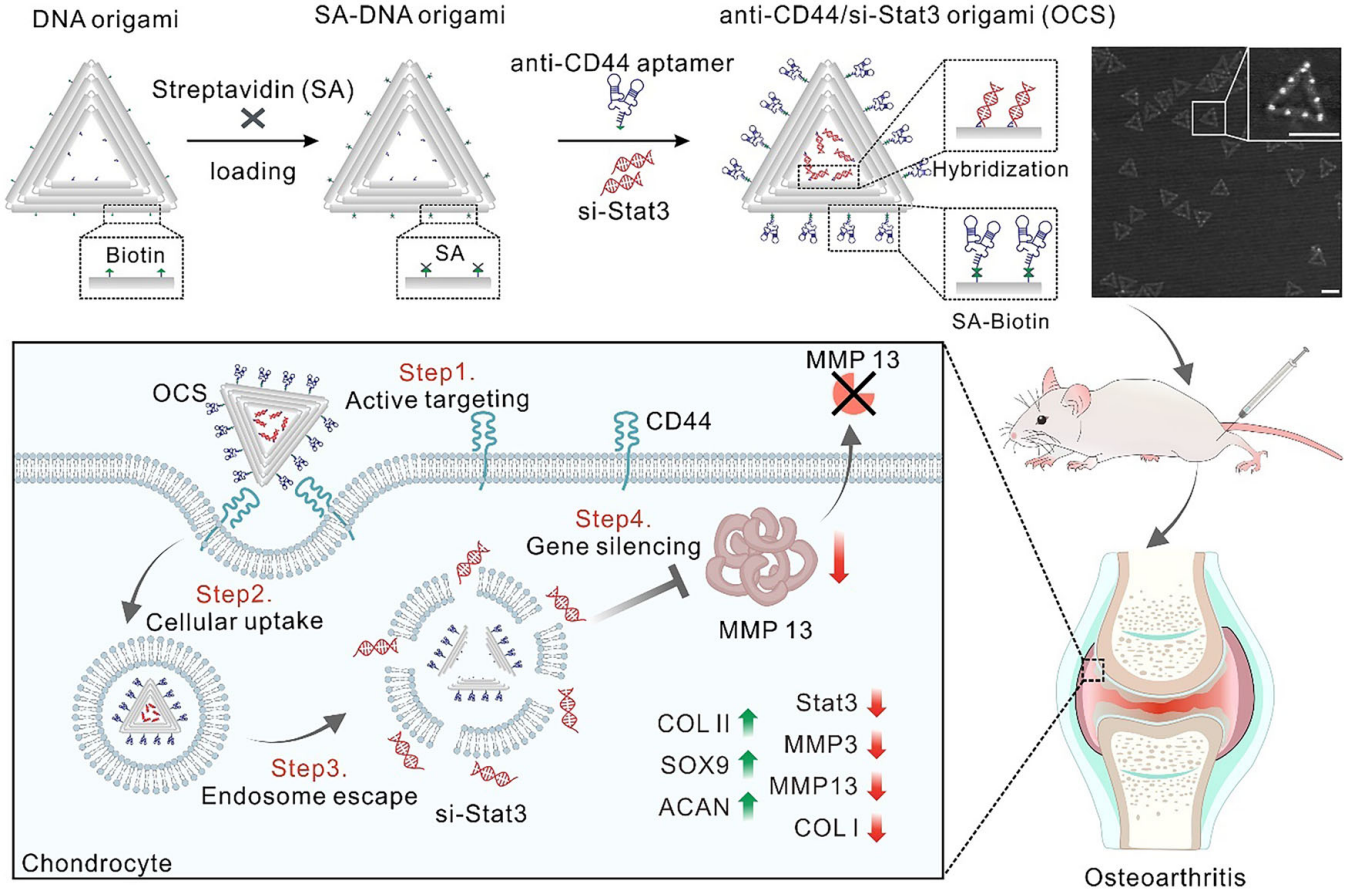
Schematic illustration of the design and construction of a DNA origami‐based chondrocyte‐targeted delivery system (OCS) for in vivo OA treatment. OCS was injected into the knee joint of OA rats, where it specifically targeted chondrocytes with high CD44 expression. This facilitated the selective recruitment of OCS by the diseased chondrocytes, promoting its internalization and enabling the effective release of si‐Stat3. As a result, Stat3 gene silencing was achieved, leading to the downregulation of MMP13 expression, a significant reduction in crtilage degradation biomarkers, and an increase in the expression of anabolic factors. Upper‐right corner: AFM image of OCS. Scale bar: 100 nm. Diagram of osteoarthritis adapted from Liu et al. Signal Transduct Target Ther. 8, 138 (2023), under a CC BY 4.0 license.

To fabricate multifunctional DNA origami‐based delivery system, we adopted a triangular DNA origami (edge length, ≈120 nm)^[^ ^43^ ^]^ including six single‐stranded poly‐T strands and twelve biotins, with precisely organized positions for the attachment of si‐Stat3 and anti‐CD44 aptamer, respectively (**Figure** **1**; Figure S1, Supporting Information). The si‐Stat3 with the end of single‐stranded poly‐A strands can be easily recruited and loaded on DNA origami by pre‐designed DNA hybridization. The biotinylated anti‐CD44 aptamers were anchored to the pre‐determined biotin sites on DNA origami using streptavidin as linkers. In this design, each SA‐binding site can anchor three biotinylated anti‐CD44 aptamers. The successful synthesis of the DNA origami‐based chondrocyte‐targeted delivery system (OCS) was characterized by atomic force microscopy (AFM) imaging and agarose gel (AGE) analysis. AFM imaging indicated the correct assembly of anti‐CD44 aptamer with the increased cross‐sectional height at pre‐designed positions (Figure S2A,B, Supporting Information). Statistical analysis of AFM images showed that 72.4% of the structures displayed 9–12 bright spots, consistent with the expected anti‐CD44 aptamer distribution (Figure S2C, Supporting Information). AGE analysis further verified the conjugation of both si‐Stat3 and anti‐CD44 aptamers to the DNA origami through distinct mobility shifts (Figure S3, Supporting Information). In additional, both the Annexin V‐FITC/PI double staining and the CCK8 assay showed almost no cytotoxicity of OCS to chondrocytes (Figure S4, Supporting Information), indicating their excellent biocompatibility. The above results indicated the successful synthesis of OCS as well as its favorable safety and stability profiles.

### 2.2 Cellular Uptake and Therapeutic Efficacy of OCS in the Cellular OA Model

**Figure 2:**
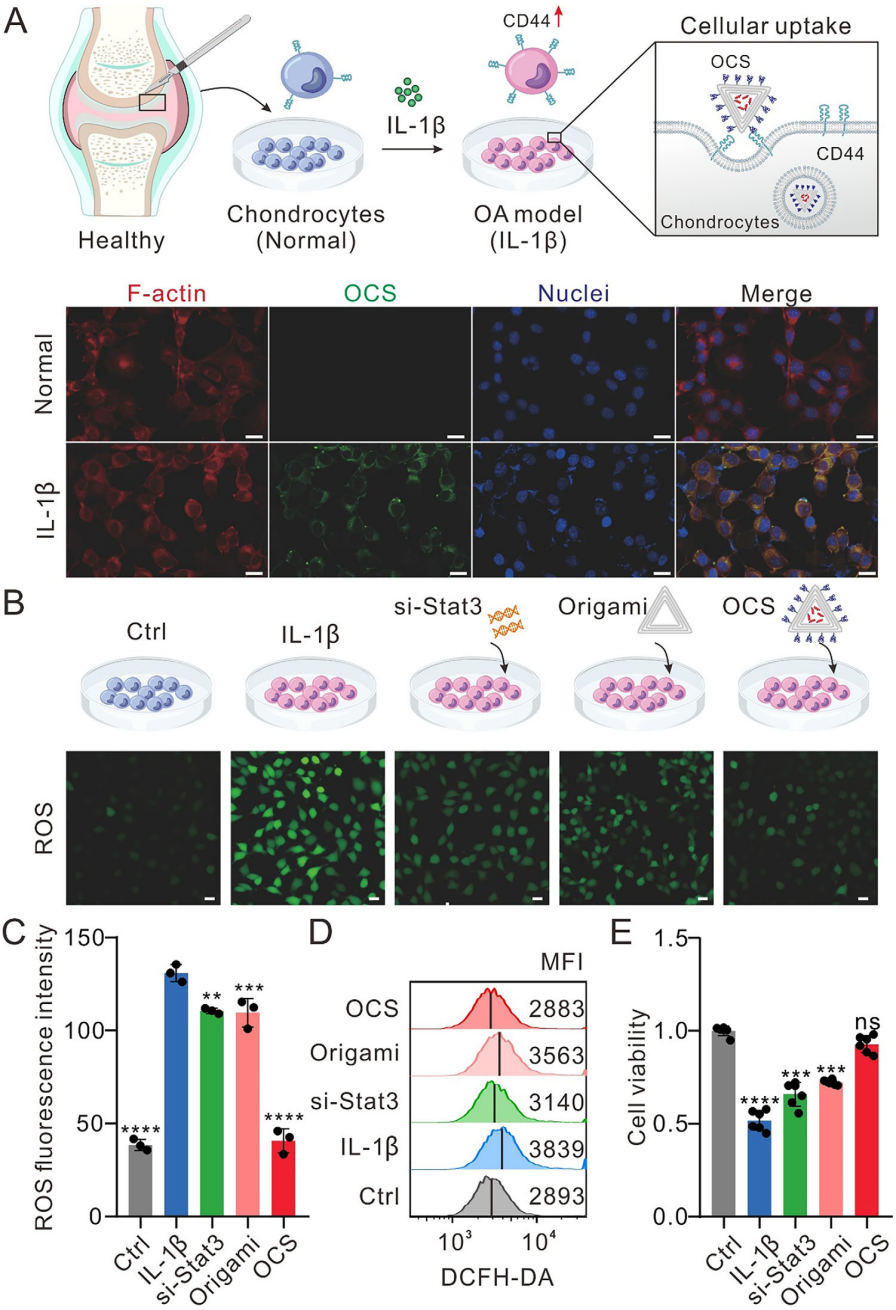
The cellular uptake, antioxidant properties, and cytoprotective effects of OCS. A) Schematic illustration and representative confocal images of OCS cellular uptake in chondrocytes under normal and inflammatory conditions (induced by IL‐1β). The cytoskeleton is labeled with phalloidin (red), OCS is labeled with FITC (green), and the nuclei were counterstained with DAPI (blue). Scale bar: 20 µm. B) Schematic and representative confocal images of ROS expression levels in OA chondrocytes after 24 h of incubation with si‐Stat3, origami, and OCS. Ctrl group: healthy chondrocytes with no IL‐1β induction or treatment; IL‐1β group, IL‐1β‐induced chondrocytes. C) Relative fluorescence intensity analysis of ROS expression levels in chondrocytes after different treatments. Data were presented as means ± SD (*n* = 3). D) Flow cytometry analysis of ROS levels in chondrocytes after different treatments. E) Cell viability of chondrocytes after 24 h of different treatments assessed by the CCK‐8 assay. Data were presented as means ± SD (*n* = 6). Statistical comparisons were performed using one‐way ANOVA. ^**^ *p* < 0.01, ^***^ *p* < 0.001, ^****^ *p* < 0.0001, ns indicates no statistical significance. Diagram of osteoarthritis (A) adapted from Liu et al. Signal Transduct Target Ther. 8, 138 (2023), under a CC BY 4.0 license.

We next investigated the cellular uptake and intracellular trafficking of DNA origami‐based chondrocyte‐targeted delivery system on the cellular OA model. To mimic the OA condition in vitro, the primary chondrocytes were co‐cultured with IL‐1β (10 ng mL^−1^), which is a key factor in promoting joint inflammation and cartilage degradation.^[^ ^44^, ^45^, ^46^ ^]^ This induction leads to OA development, resulting in an upregulation of CD44 expression on the surface of the affected chondrocytes. The cellular uptake efficiency of OCS labeled with fluorescein isothiocyanate (FITC) was observed in normal and inflammatory (IL‐1β induced) conditions using confocal laser scanning microscopy (CLSM). The results showed that the anti‐CD44 aptamer effectively guides OCS to specifically bind to and enter chondrocytes under the condition of IL‐1β‐induced degeneration, while was nearly unable to enter healthy chondrocytes (**Figure** **2**A). Time‐dependent localization analysis revealed that OCS initially accumulated near the membrane with low lysosomal colocalization at 0.5 h (Pearson's coefficient = 0.15), peaked in lysosomes at 6 h (0.62), and successfully escaped into the cytoplasm by 12 h (0.26), indicating effective endosomal escape (Figure S5, Supporting Information).

We further evaluated the therapeutic capability of OCS on the cellular OA model, including ROS measurement and apoptosis analysis. Previous studies determined that elevated ROS levels will lead to chondrocyte apoptosis and cartilage degradation. IL‐1β treatment significantly increased ROS production compared to healthy chondrocytes (denoted as control group), while OCS reverted the ROS level back to the level of healthy chondrocytes, and si‐Stat3 or DNA origami is less effectively (Figure 2B–D). This pronounced reduction highlights the multifunctional miRNA delivery system in lowering ROS levels, which is beneficial for mitigating oxidative stress and protecting chondrocytes from apoptosis. As expected, Similar results were observed in chondrocyte proliferation using the CCK8 assay. OCS could full recovery the chondrocyte proliferation injured by IL‐1β comparing with si‐Stat3, DNA origami (Figure 2E). These results suggested that OCS has significant therapeutic potential at the cellular level, offering a promising new avenue for the effective management of osteoarthritis.

### 2.3 OCS Enhances Cartilage Protection and Synthesis in an In Vitro OA Model

**Figure 3:**
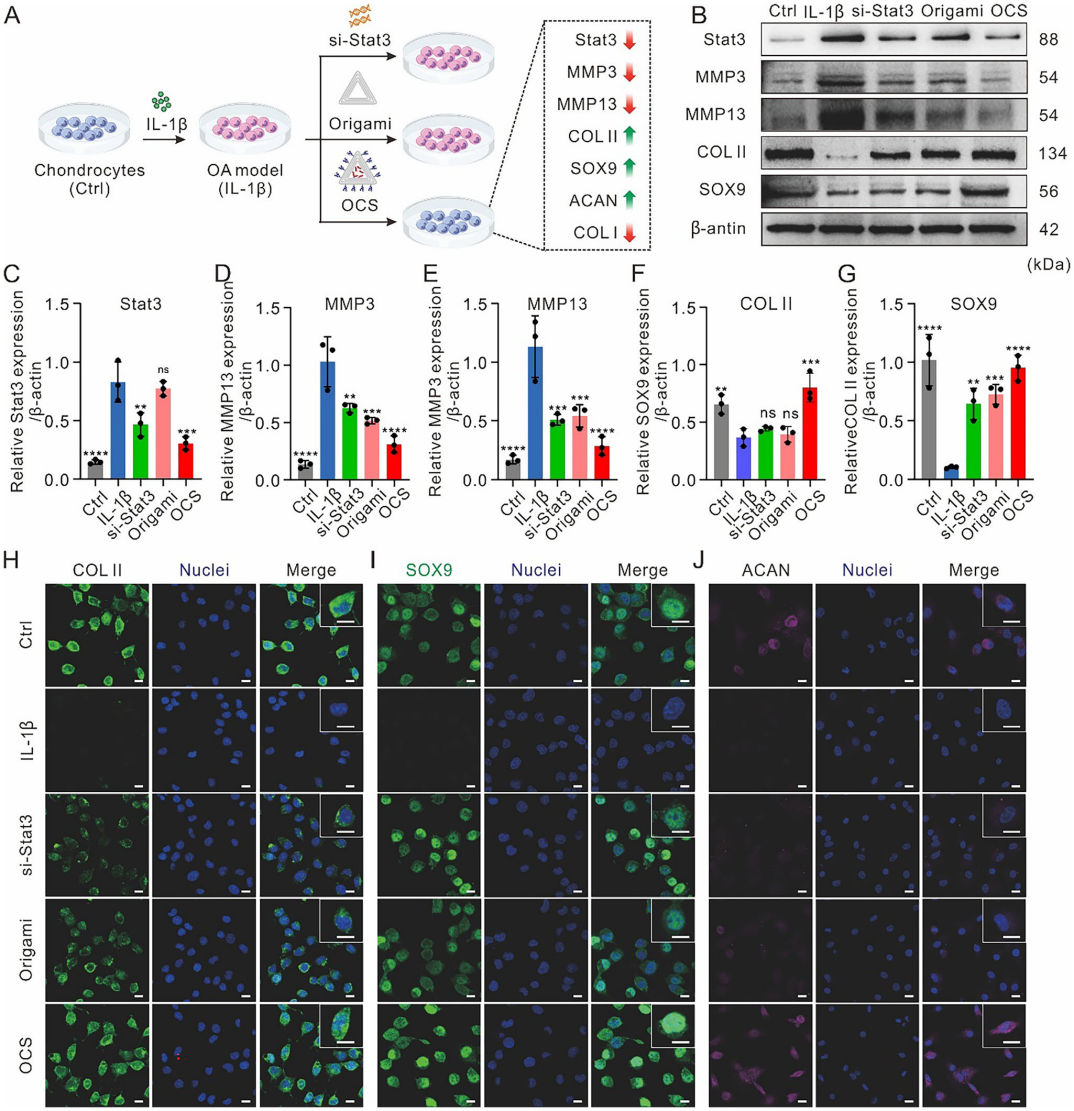
OCS modulates catabolic and anabolic proteins in OA chondrocytes. A) Schematic illustration of the cellular OA model establishment in rat chondrocytes by IL‐1β induction, followed by treatment with si‐Stat3, Origami, and OCS. Ctrl group: healthy chondrocytes with no IL‐1β induction or treatment; IL‐1β group, IL‐1β‐induced chondrocytes. B) Western blot analysis of Stat3, MMP3, MMP13, COL‐II, and SOX9 protein expression in chondrocytes after different treatments. C–G) Quantitative analysis of the western blot results. Data were presented as means ± SD (*n* = 3). Statistical comparisons were performed using one‐way ANOVA. ^**^ *p* < 0.01, ^***^ *p* < 0.001, ^****^ *p* < 0.0001, ns indicates no statistical significance. H–J) Representative Immunofluorescence images of the protein expression of COL‐II, SOX9, and ACAN in chondrocytes after different treatments. Scale bar: 20 µm.

We further investigated the cartilage protective effects of DNA origami‐based chondrocyte‐targeted si‐Stat3 delivery system by treating IL‐1β‐induced chondrocytes isolated from rat intra‐articular tissues (**Figure** **3**A). Stat3, a key transcription factor mediating inflammation, is upregulated by IL‐1β. Previous studies have shown that targeting Stat3 with siRNA reduces MMP13 activity, mitigating cartilage degradation and promoting preservation.^[^ ^47^, ^48^, ^49^ ^]^ To assess the therapeutic effects, we performed western blot (WB) analysis to quantify the expression of cartilage degradation markers (Stat3, MMP3, MMP13) and synthesis markers (COL‐II, SOX9) (Figure 3B). As expected, IL‐1β induction substantially upregulated Stat3, MMP3, and MMP13 compared to the control group. Treatments with si‐Stat3, Origami, and OCS in IL‐1β‐induced chondrocytes reduced inflammation, as evidenced by decreased Stat3, MMP3, MMP13 level. Notably, OCS exhibited the most potent inhibitory effect (Figure 3C–E). In contrast, low expression of chondrogenesis‐related proteins, including COL‐II and SOX9, was observed in IL‐1β‐induced chondrocytes. COL‐II, a key extracellular matrix (ECM) component, supports chondrocytes in articular cartilage, while SOX9, a critical transcription factor, maintains the chondrocyte phenotype. The expression of COL‐II and SOX9 was higher in the si‐Stat3, Origami, and OCS treatment groups compared to the IL‐1β group, with OCS showing the most pronounced effect (Figure 3F,G). Immunofluorescent (IF) staining confirmed the upregulation of COL‐II and SOX9 secretion following treatment, particularly with OCS (Figure 3H,I; Figure S6A,B, Supporting Information). Similarly, aggrecan (ACAN), another ECM component essential for cartilage function, was upregulated (Figure 3J; Figure S6C, Supporting Information). Furthermore, type I collagen (COL‐I), a marker of fibrotic changes and cartilage degeneration in OA, was downregulated in all treatment groups, especially OCS, suggesting that OCS promotes ECM accumulation and prevents fibrosis (Figure S7, Supporting Information). These results demonstrate that OCS treatment effectively mitigates IL‐1β‐induced cartilage damage, promotes anabolic metabolic factors in chondrocytes, and restores homeostasis of articular cartilage compared to other therapeutic approaches.

### 2.4 Biodistribution and Biosafety of OCS

**Figure 4:**
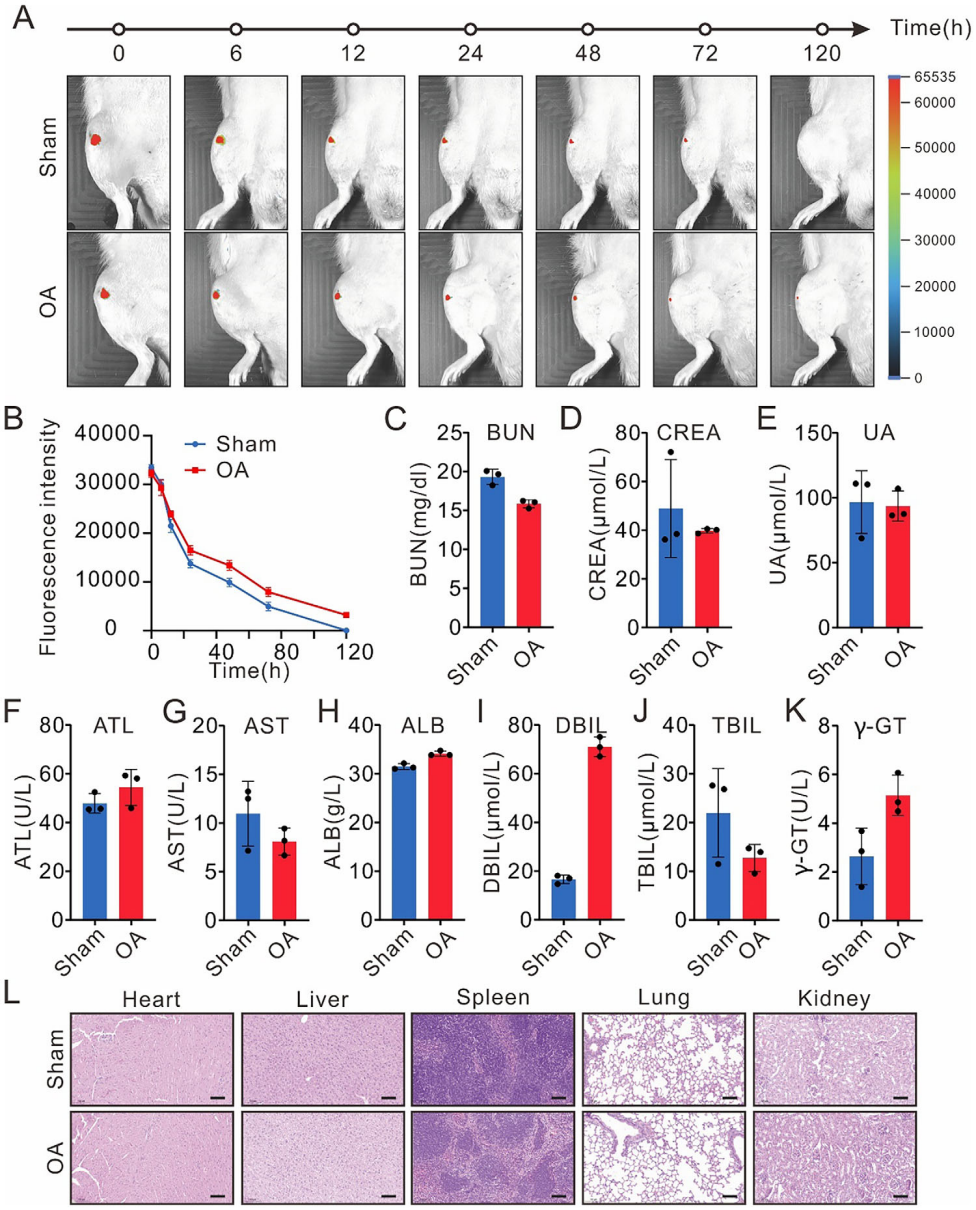
In vivo distribution and biosafety of OCS in rats. A) Representative in vivo fluorescence images of the knee joints in Sham and OA rats at various time points following intraarticular injection with FITC‐OCS (*n* = 3 per group). B) Quantitative analysis of fluorescence radiation in (A). C–E) Blood biochemical indicators of renal function, BUN: blood urea nitrogen, CREA: creatinine, UA: uric acid. Data were presented as means ± SD (*n* = 3). F–K) Liver function test indexes of rats. ATL: alanine transaminase, AST: aspartate transaminase, ALB: albumin, DBIL: direct bilirubin, TBIL: total bilirubin, γ‐GT: gamma‐glutamyl transferase. Data were presented as means ± SD (*n* = 3). L) Representative H&E staining images of the major organs from both OA and Sham groups of rats. Scale bar: 100 µm.

Targeted accumulation at disease sites with minimal impact on healthy tissues is essential for maximizing therapeutic efficacy and biosafety. To visualize the distribution and retention of OCS in knee joints, we labeled the staple strands with FITC and intra‐articularly injected the FITC‐labeled OCS into both Sham and OA rats. For the OA model in mice, we performed anterior cruciate ligament transection (ACLT) and partial medial meniscectomy (pMMx) on the right hind limb knee, a well‐established surgical method for inducing OA. We performed in vivo imaging of the knee joint at seven time points (0, 6, 12, 24, 48, 72, and 120 h) following intra‐articular injection of FITC‐labeled OCS. The imaging revealed higher fluorescence intensity in OA rat joints compared to sham groups at all time points. Notably, OCS retention in OA joints was prolonged, lasting up to 120 h, whereas retention in the sham joints was relatively short (**Figure** **4**A,B). After 120 h, ex vivo fluorescence imaging of major organs confirmed this prolonged retention specifically in the OA joints (Figure S8, Supporting Information). Importantly, no OCS retention was observed in metabolic organs such as liver and kidney in either Sham or OA rats, indicating precise targeting and localization of OCS to the knee joints. These results highlight the extended retention of OCS, which may enhance treatment efficacy. To validate CD44‐mediated targeting in vivo, FITC‐labeled OCS (with anti‐CD44 aptamers) and non‐targeted OC (without anti‐CD44 aptamers) were injected into OA joints. Cryosection analysis at 24 h post‐injection showed clear cytoplasmic fluorescence in the OCS group, while the minimal signal was detected in the OC group, confirming the aptamer‐driven targeting specificity (Figure S9, Supporting Information).

To evaluate the biosafety of OCS, we investigated its potential to induce liver or kidney damage. Several biomarkers associated with liver and kidney function were measured and found to be within normal physiological ranges in both sham and OA rats following OCS administration. These included urea nitrogen (UN), creatinine (CREA), and uric acid (UA) for kidney health (Figure 4C–E), and alanine aminotransferase (ALT), aspartate aminotransferase (AST), albumin (ALB), direct bilirubin (DBIL), total bilirubin (TBIL), and gamma‐glutamyl transferase (γ‐GT) for liver function (Figure 4F–K). Similarly, a blood routine analysis showed no abnormalities, with all parameters remaining within normal levels (Figure S10, Supporting Information). Histological examination of the liver and kidneys revealed no significant pathological changes following OCS administration. Additionally, no abnormalities were observed in other major organs, including the heart, spleen, and lungs, further confirming the biosafety of the DNA origami‐based delivery system (Figure 4L). These results demonstrate that the DNA origami‐based delivery system does not induce liver or kidney toxicity, supporting its favorable safety profile for therapeutic use.

### 2.5 OCS Triggers Robust Therapeutic Efficacy In Vivo

**Figure 5:**
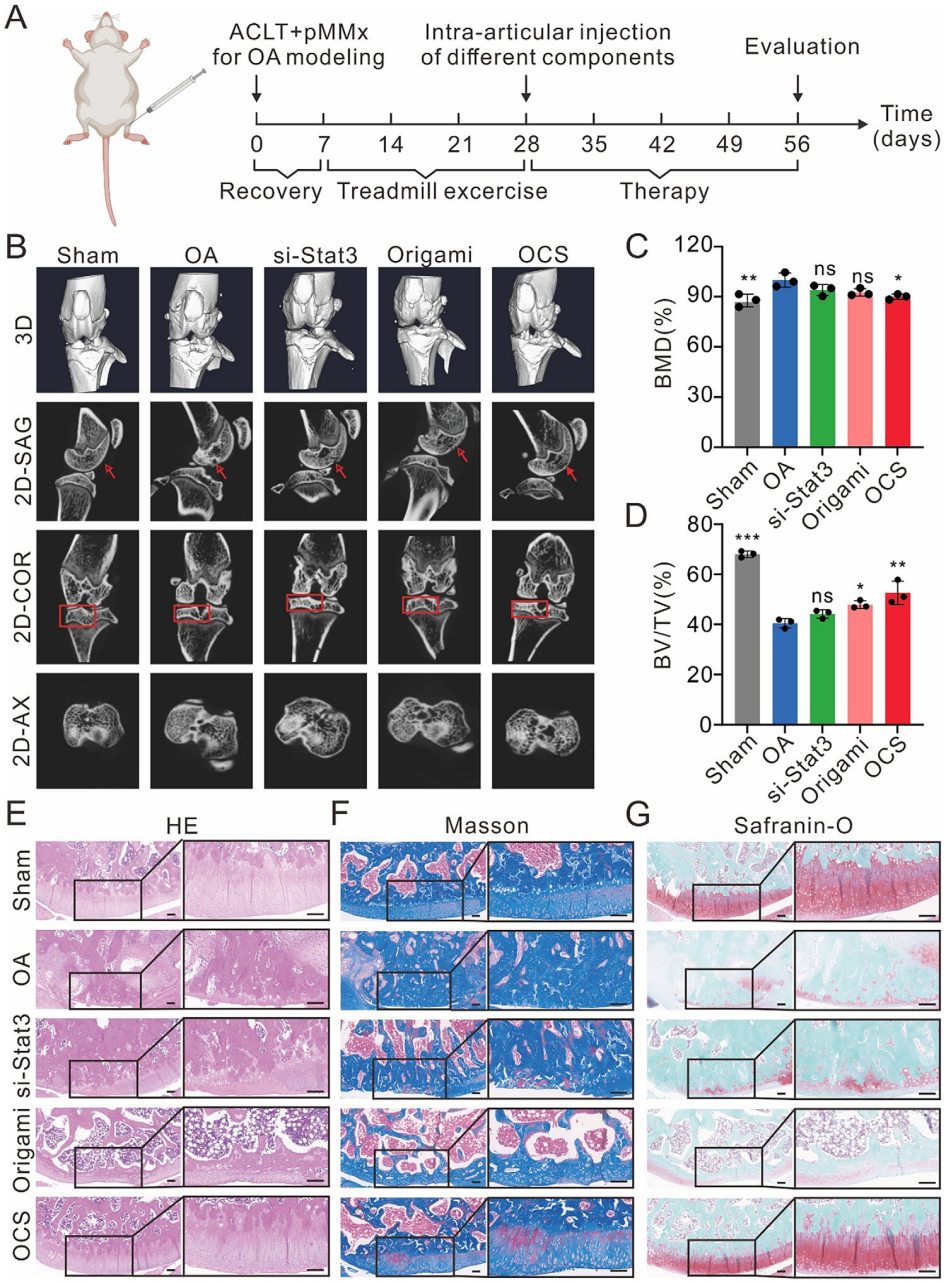
Therapeutic effects of OCS on articular cartilage in a rat OA model. A) A schematic diagram illustrating the experimental design. An OA model was established using the ACLT and pMMx surgical technique. After four weeks, intra‐articular injections of normal saline, si‐Stat3, origami, or OCS were administered weekly, totaling four injections. At the ninth week, cartilage recovery was evaluated using micro‐CT imaging and histological staining. Sham group: only the skin and muscles were separated, leaving the joint structures intact. OA group: treated with normal saline. B) Representative 3D micro‐CT images of the knee and 2D images in the coronal (COR), sagittal (SAG), and axial planes (AX). C,D) Quantitative analysis of tibial subchondral bone parameters: bone mineral density (BMD) and volume/tissue volume (BV/TV). Data were presented as means ± SD (*n* = 3). Statistical comparisons were performed using one‐way ANOVA. ^*^ *p* < 0.05, ^**^ *p* < 0.01, ^***^ *p* < 0.001, ^****^ *p* < 0.0001, ns indicates no statistical significance. E–G) Histological analyses of the knee joint, including H&E, Masson, and Safranin O staining. Photographs of the right are higher magnification of the black rectangular areas. Scale bars: 200 µm.

We next evaluated the therapeutic efficacy of OCS in OA rats following the treatment regimen outlined in **Figure** **5**A. Briefly, rats underwent either sham surgery or ACLT and pMMx surgery to induce OA in the knee joints. After one week of postoperative recovery, the rats were subjected to three weeks of cycling exercise. OA rats then received intra‐articular injections of normal saline, si‐Stat3, origami, or OCS one time per week for four weeks. At 8 weeks post‐surgery, micro‐computed tomography (micro‐CT) and histological analyses of knee joint sections were performed to assess knee joint damage (Figure 5A). As expected, micro‐CT analysis showed that, compared to the sham group, OA group (treated with normal saline) exhibited significant remodeling of subchondral bone, characterized by cortical thickening, increased bone mineral density (BMD), and a reduced bone volume to tissue volume ratio (BV/TV). In contrast, the subchondral bone changes in rats treated with OCS group were mild, indicating that OCS injection helps protect the subchondral bone from structural alterations and reduces bone mineralization density (Figure 5B–D). The therapeutic effects of si‐Stat3 and origami were less pronounced, further underscoring the superior efficacy of OCS in mitigating bone damage.

Histological analysis, including H&E, Masson, and Safranin‐O staining, was performed to evaluate the morphological characteristics of cartilage and bone tissues. In the OCS treatment group, substantial improvements were observed, including reduced cartilage damage, thinner synovial membranes, decreased fibrosis, and preservation of cartilage collagen, compared to the OA group. High‐magnification images highlighted these therapeutic effects, demonstrating superior cartilage preservation achieved in OCS group (Figure 5E–G). Complementary histological assessments, including Mankin's scores (Figure S11A, Supporting Information) and OARSI grades (Figure S11B, Supporting Information), confirmed the significant protective effects of the OCS treatment on cartilage integrity. Consistent with the in vitro results, OCS demonstrated greater therapeutic efficacy in OA than si‐Stat3 and origami.

**Figure 6:**
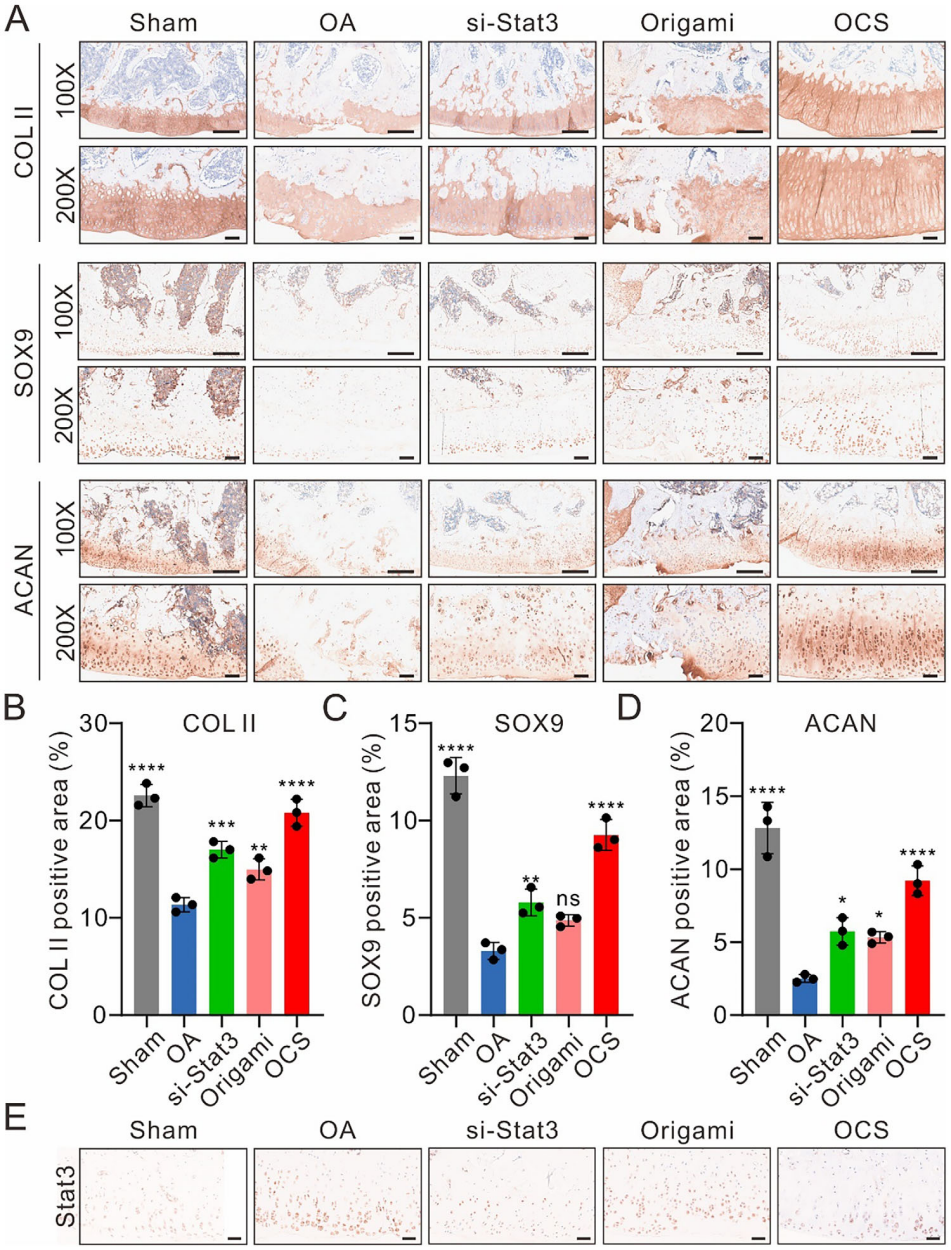
Immunohistochemical (IHC) staining of knee joints from rats in different treatment groups of rats after four weeks of treatment. A) Representative IHC staining images of CoL‐II, SOX9, and ACAN in rat cartilage sections under different treatment conditions. Scale bar: 500 µm. B) The CoL II‐positive (CoL II^+^) area is calculated from(A). C) The SOX9‐positive (SOX9^+^) area is calculated from (A). D) The ACAN‐positive (ACAN^+^) area is calculated from (A). Data were presented as means ± SD (*n* = 3). Statistical comparisons were performed using one‐way ANOVA. ^*^ *p* < 0.05, ^**^ *p* < 0.01, ^***^ *p* < 0.001, ^****^ *p* < 0.0001, ns indicates no statistical significance. E) Representative IHC staining images of Stat3 in rat cartilage sections under different treatment conditions. Scale bar: 100 µm.

To investigate the chondrocyte phenotype in OA rats under different treatment conditions, we performed immunohistochemical staining for ACAN, COL‐II, and SOX9. In the sham group, these cartilage markers exhibited distinct distribution patterns within the cartilage tissue: SOX9 was primarily localized in the nucleus, COL‐II was predominantly found in the cytoplasm, and ACAN showed variable distribution at different cartilage depths (**Figure** **6**A). As expected, these characteristics were disrupted in the OA group. Compared to the OA group, rats treated with si‐Stat3, origami, and OCS showed higher density and continuity of expression for ACAN, COL‐II, and SOX9. Notably, the staining patterns of COL‐II and ACAN in the OCS treatment group closely resembled those of the sham group. Dense SOX9‐positive granules, appearing as brownish‐yellow particles within the chondrocyte nuclei, were observed in the cartilage, indicating that OCS treatment had the most favorable effect on cartilage regeneration (Figure 6A–D). Furthermore, immunohistochemical analysis of Stat3 revealed that both the si‐Stat3 and OCS treatment groups effectively reduced Stat3 expression, which in turn decreased extracellular matrix degradation and inflammation (Figure 6E; Figure S12, Supporting Information). These findings suggest that the multivalent OCS platform significantly promotes cartilage regeneration and alleviates the progression of osteoarthritis.

## 3 Conclusion

In summary, we developed a DNA origami‐based chondrocyte‐targeted delivery system for siRNA with nanometric precision to enhance therapeutic efficacy in OA. This system offers several key advantages for OA treatment. On the one hand, the triangular DNA origami structure adopted from a well‐established design, was selected for its rapid assembly, structural stability, and efficient cargo loading. Its geometry also facilitates the precise spatial organization of targeting ligands (anti‐CD44 aptamers) and therapeutic agents (si‐Stat3), optimizing interactions with specific cell surface receptors and enabling controlled release. This targeted delivery improves cellular uptake while minimizing off‐target effects commonly seen in traditional drug delivery systems. On the other hand, our findings demonstrate that si‐Stat3, a critical regulator of inflammation and chondrocyte apoptosis in OA, is effectively delivered via DNA origami, resulting in significant reductions in inflammation and preservation of cartilage. Furthermore, even without specific targeting, DNA origami itself exhibited therapeutic effects, which may be due to its inherent stability, biocompatibility, and potential to interact with cellular components. Previous studies have also reported similar findings, suggesting that the materials alone may possess intrinsic therapeutic properties.^[^ ^50^, ^51^, ^52^ ^]^ However, further research is needed to fully understand the mechanisms behind these effects. These results underscore the dual functionality of DNA origami: while targeted delivery enhances therapeutic outcomes, its intrinsic properties provide additional benefits, making it a versatile platform for OA therapy and broader nanomedicine applications.

Despite these promising results, several limitations remain. Experiments conducted in vitro and in vivo using rat models, while insightful, do not fully replicate the complexity of human OA. Further research in advanced animal models and clinical trials is necessary to validate the efficacy and safety of this approach in humans. Additionally, scaling the production of CD44‐targeted DNA origami for clinical use poses challenges due to the precision required in synthesis and modification. Long‐term safety and potential immune responses in humans also require further exploration.

DNA origami holds immense potential in drug delivery, with numerous exciting applications yet to be explored. Its tunable architecture enables customization for a wide range of therapeutic agents, opening new avenues for targeted treatments of inflammatory diseases and cancers.^[^ ^53^, ^54^, ^55^, ^56^ ^]^ For instance, dual‐responsive DNA origami systems have been designed for both imaging and treatment of sepsis‐associated acute kidney injury,^[^ ^57^ ^]^ while DNA origami‐based enzymatic nanoreactors have been shown to enhance antitumor immunity through spatially coordinated catalytic reactions.^[^ ^58^ ^]^ In parallel, other DNA nanostructures such as DNA frameworks (e.g., tFNA) have demonstrated therapeutic potential by improving targeted delivery in complex immune environments via mechanisms like neutrophil hitchhiking and aptamer multimerization, thereby enhancing anti‐inflammatory efficacy.^[^ ^59^, ^60^, ^61^ ^]^ Furthermore, the programmability and versatility of DNA nanotechnology offer exciting opportunities for designing advanced molecular sensors and smart drug delivery systems, which could promote personalized medicine.^[^ ^62^ ^]^ Given its modularity, DNA origami could also holds promise in creating multi‐functional platforms for combination therapies, enhancing treatment efficacy in complex diseases, and unlocking further innovative applications in drug delivery and nanomedicine, thereby expanding its therapeutic potential.^[^ ^63^ ^]^

## 4 Experimental Section

### Extraction and Incubation of Chondrocytes

Primary chondrocytes were obtained from the knee joint cartilage of neonatal SD rats. After mechanical disruption and enzyme treatment, cartilage fragments underwent centrifugation at 350 × g for 10 min to isolate the chondrocytes. The primary chondrocytes were maintained using the iCell primary chondrocyte culture system (Primed‐iCell‐020, iCell, China), with 10% FBS and 1% penicillin‐streptomycin (Invitrogen) added, and incubated at 37 °C under a 5% CO_2_ environment. For the experiments conducted, chondrocytes from passages 1 to 4 were utilized.

### Animal Experiments

All rats were raised and cared according to the guidelines on Laboratory Animals of Shanghai First People's Hospital with the production license number of 2024AWS470. All animal procedures were approved by the Laboratory Animal Management Committee of Shanghai First People's Hospital. SD rats aged between 6 and 8 weeks, with body weights ranging from 200 to 220 g, were procured from Beijing Vital River Laboratory Animal Technology Co., Ltd.

### Design, Fabrication, and Characterization of OCS

M13mp18 bacteriophage DNA was obtained from Bayou Biolabs (Shanghai, China), and DNA oligonucleotides were Purchase from Sangon Biotech (Shanghai, China). The triangular DNA origami structure was designed with strategically positioned oligonucleotide overhangs, including 12 externally biotin‐modified overhangs employed for CD44‐targeted tagging and 6 internal overhangs for si‐Stat3 binding. The assembly of the biotinylated DNA origami was performed by heating a mixture of M13mp18 DNA and short staple DNA in a buffer, referred to as TAE‐Mg^2^⁺, at 95 °C for 5 min, followed by a gradual cooling process from 95 to 25 °C using a PTC‐200 Peltier Thermal Cycler. Unbound staple DNA strands were removed through purification using Millipore Amicon Ultra 100 kDa spin columns. After co‐incubation of the synthesized triangular origami with SA (S9171, Solarbio, China) for 30 min at room temperature, biotinylated anti‐CD44 DNA aptamer and si‐Stat3 were added for overnight incubation at 4 °C. The obtained OCS was quantified via UV spectrophotometry (absorption at 260 nm) after being purified and stored at 4 °C for subsequent use.

### Agarose Gel Electrophoresis (AGE) Assay

The M13mp18 scaffold, unmodified triangular DNA origami, streptavidin (SA)‐conjugated origami, anti‐CD44 aptamer‐modified origami, and the final construct containing both anti‐CD44 aptamers and si‐Stat3 were identified through 1% agarose gel electrophoresis in 1×TAE‐Mg^2+^ buffer at 90 V. Following the process, the gel was subjected to UV light exposure and the DNA bands were analyzed with quantify software from Tanon Science & Technology Co., Ltd. located in China.

### Atomic Force Microscope (AFM) Imaging

The triangle DNA origami and OCS were characterized with AFM. To observe with AFM, freshly assembled diluted samples (3 nm) were deposited onto the muscovite mica surface for 3 min. Subsequently, the mica surface was washed 2–3 times with distilled water to eliminate any unabsorbed samples, and then dried with high‐purity nitrogen gas. The obtained samples were tested with ScanAsyst‐Air tips at a spring constant of 0.4 N/m under ScanAsyst Mode at a resolution of 512 pixels per line and a scan rate of 2 Hz.

### Primary Chondrocytes Uptake of OCS

Chondrocytes were plated at a density of 4 × 10^4^ cells per well in confocal culture dishes and were incubated with IL‐1β (10 ng mL^−1^) for 24 h to trigger an inflammatory response, establishing an OA cell model, while normal chondrocytes were not subjected to this treatment. Subsequently, both normal and OA cell models were co‐cultured with 20 nm FITC‐labeled OCS for 6 h. To directly observe the internalization of OCS, confocal microscopy (Nikon, Japan) was employed. Following fixation with 4% paraformaldehyde, chondrocytes were sequentially stained with TRITC‐labeled cycloheximide and the nuclei were stained with 4′,6‐diamidino‐2‐phenylindole (DAPI).

### Lysosomal Escape Experiment

Chondrocytes (1 × 10^6^ cells) were seeded in confocal dishes. After allowing the cells to adhere, FITC‐labeled OCS (20 nm) was added, and incubated for 0.5, 6, and 12 h. At these three time points, a pre‐warmed lysosomal probe (75 nm, 40739ES50, YEASEN, China) was added at 37 °C and incubated for 1 h. The cells were then washed twice with PBS for 5 min each. Following this, DAPI (5 µg mL^−1^) was added for 10 min, and the cells were washed again with PBS twice for 5 min each. Finally, seal the film with 50% glycerol and perform confocal microscopy imaging.

### Cell Viability Assay

To assess the cellular safety and protective effects of OCS on chondrocytes, the Enhanced CCK‐8 (C0042, Beyotime, China) were utilized to determine cell viability. A total of 3 × 10^3^ primary chondrocytes per well were placed in a 96‐well plate and cultured overnight. After adherence, chondrocytes were treated with varying concentrations of OCS for 24 h to conduct toxicity assays. For cell‐protective experiments, the control group was not treated, and the OA cell models were divided into IL‐1β group and three experimental groups. The experimental groups received specified treatments (si‐Stat3, Origami, and OCS) at designated doses, followed by a 24 h incubation to evaluate the protective effects of OCS. Following a 4 h incubation with CCK‐8 reagent, the effect was determined by measuring and analyzing the absorbance at 450 nm.

### ROS Detection

Chondrocytes were divided into five groups according to different treatment factors: Ctrl, IL‐1β, si‐Stat3, Origami, and OCS. After a 24 h incubation period, intracellular ROS levels were assessed employing a ROS Assay Kit (S0033S, Beyotime, China). The cells were treated with the DCFH‐DA probe at a 1:1000 dilution and incubated for 20 min at 37 °C to allow the probe to penetrate and react. After incubation, a confocal laser scanning microscope (Nikon, Japan) was employed to capture images for further analysis.

### Flow Cytometry

Flow cytometry was employed to assess the effects of OCS on apoptosis and ROS production in chondrocytes. Chondrocytes (4 × 10^5^ cells mL^−1^) were seeded into six‐well plates and incubated for 24 h, after which they were treated with IL‐1β to establish a chondrocyte OA model. Subsequently, the cells were treated for an additional 24 h with Ctrl (chondrocyte medium), IL‐1β, si‐Stat3, Origami, and OCS. After treatment, the cells were digested, and apoptosis and ROS expression were measured using dedicated detection kits according to the manufacturer's instructions. Data collection for all samples was conducted using the Attune NxT flow cytometer (Thermo Scientific, USA).

### Western Blot

Chondrocytes were seeded into five 10 cm culture dishes, divided into the following groups: Ctrl (chondrocyte medium), IL‐1β, si‐Stat3, Origami, and OCS. The cells were first treated with IL‐1β for 24 h, followed by the respective treatments. After digestion, total protein was extracted from the cells in each group using a Kit (KGB5303, Keygen Biotech, China), and protein concentrations were determined using a BCA Protein Assay Kit (P0012, Beyotime, China). After mixing the samples with 5× loading buffer at a 4:1 ratio, the mixture was heated to 100 °C for 10 min. Target markers were separated into proteins of their corresponding molecular weights by SDS‐PAGE using a 10% gel. The proteins were then transferred to a PVDF membrane (Bio‐Rad, USA) and incubated in Ncm Blocking Buffer (P30500, Ncm, China) for 30 min. The membrane was incubated with primary antibodies overnight at 4 °C (SOX9, MMP3, MMP13, COLII, Stat3, and β‐actin) (Proteintech, China). The following day, after re‐heating, the membrane was immersed in a secondary antibody solution (HRP‐conjugated Affinipure Goat Anti‐Rabbit IgG) (Proteintech, China) for 1 h and washed with TBST (ST673, Beyotime, China). Finally, the target protein bands were visualized using BeyoECL Plus (P0018S, Beyotime, China) and analyzed with ImageJ 1.53c.

### Immunofluorescence Staining

Following the application of various treatments (Ctrl, IL‐1β, si‐Stat3, Origami, and OCS), the chondrocytes were rinsed three times with PBS. The cells were thoroughly rinsed before fixation in 4% paraformaldehyde for 30 min. To permeabilize the membrane, 0.3% Triton X‐100 was applied, followed by blocking with 5% normal goat serum to minimize nonspecific binding. The primary antibody was applied to the chondrocytes and incubated overnight at 4 °C. Afterward, the primary antibody was replaced with the corresponding secondary antibody, and incubation proceeded for 1 h at room temperature. The cells underwent DAPI staining, and imaging was conducted with a confocal laser scanning microscope (Nikon, Japan). The acquired images were analyzed using ImageJ 1.53c software.

### Biosafety Assessment

To assess the biosafety of OCS, six to eight‐week‐old SD rats (200–220 g) were classified into two groups including normal and OA groups (*n* = 3 per group). Following intraarticular injection of FITC‐OCS into the knee joint of the rats, in vivo, imaging was performed at 0, 6, 12, 24, 48, 72, and 120 h using the FUSION FX7 SPECTRA system (VIBER, France) to assess the retention and distribution of OCS. After 120 h, the rats were euthanized, and both the limbs and major organs were collected for analysis using a whole‐body fluorescence imaging system. Then the main organs were analyzed histologically by H&E staining. Blood samples were collected for hematological examination.

### Construction of OA Models

SD rats randomly assigned to five groups: sham, OA, si‐Stat3, Origami, and OCS (*n* =6 per group). Intraperitoneal injection of 10% chloral hydrate was used for anesthesia induction. After shaving the right knee, the area was cleaned with povidone‐iodine and 75% ethanol. The skin and muscles around the knee were carefully separated, the anterior cruciate ligament was excised, and a partial meniscectomy of the medial meniscus was performed. After cleaning the surgical site and suturing the wound, the animals received penicillin injections to reduce the risk of infection. In the sham group, only the skin and muscles were separated without damaging the joint structures. Following one week of recovery from surgery, the rats underwent daily treadmill training for 1 h over three weeks. Treatment interventions began thereafter. While the sham group received no further treatment, the OA group received intra‐articular injections of 100 µL normal saline, and the other groups were injected with their respective treatments (si‐Stat3, Origami, or OCS) three times per week. After four weeks of treatment, all rats were assessed.

### Microcomputed Tomography (Micro‐CT)

The entire right knee joint of the rat, encompassing the joint itself, the distal femur, and the proximal tibia, was carefully extracted, with all surrounding muscles and ligaments removed. After fixation in a 4% paraformaldehyde solution, the samples were prepared and positioned on the workstation stand, ensuring that the regions of interest were aligned within the scanning field of the CT device. Micro‐CT scanning was performed using the Skyscan 1174 system (operating at 50 kV and 800 µA). 3D reconstruction of the targeted regions was carried out using N‐Recon software, and bone mineral density (BMD) was analyzed with CT‐AN software.

### Histological Analysis

The knee joints were preserved in a 4% paraformaldehyde solution for 3 days, followed by decalcification in a formalin‐EDTA solution over 6 weeks. Afterward, they were dehydrated with increasing concentrations of ethanol, embedded in paraffin, and sliced into thin sections. These sections were subjected to staining with H&E, Masson, and Safranin‐O. All stained samples were scanned using a tissue scanner (Aperio, ScanScope XT, USA). Imaging and analysis of the sections were conducted under 100× and 200× magnifications using the scanner's integrated software (Aperio, Image Scope, USA). Osteoarthritis (OA) severity was assessed through histological analysis following the semiquantitative OARSI scoring system and a modified Mankin score based on histological morphology. Immunohistochemistry (IHC) was performed using primary antibodies targeting COL II, Stat3, SOX9, and AGG (Proteintech, China).

### Statistical Analysis

Each experiment was carried out independently and repeated a minimum of three times. Data were presented as mean ± SD, and the provided data represented the observed outcomes. Statistical comparisons among three or more groups were performed using one‐way ANOVA. Data analysis was conducted using GraphPad Prism software. Statistical significance was determined as follows: ^*^ *p* < 0.05, ^**^ *p* < 0.01, ^***^ *p* < 0.001, ^****^ *p* < 0.0001, ns indicates no statistical significance.

## Conflict of Interest

The authors declare no conflict of interest.

## Author Contributions

Q.L. and X.Z. contributed equally to this work and shared the first authorship. Q.L., X.Z., S.T., X.J., and Y.Z. performed experiments and data analysis. Y.Z., Y.S., and C. M. wrote the paper. H.P., Z.Y., and C.M. supervised the project and revised the paper. All the authors have read and approved the final version of this paper.

## Supporting information



Supporting Information

## Data Availability

The datasets generated during and/or analyzed during the current study are available from the corresponding author upon reasonable request.
