# Supplementary material for: DNA Origami‐Based CD44‐Targeted Therapy Silences Stat3 Enhances Cartilage Regeneration and Alleviates Osteoarthritis Progression
Source: Adv Sci (Weinh). 2025 May 21;12(29):e03939. doi: 10.1002/advs.202503939 (PMC12362795; doi:10.1002/advs.202503939)
Supplement: Supplementary file 1 — Supporting Information [file ADVS-12-e03939-s001.docx]

Supporting Information

**DNA Origami-Based CD44-Targeted Therapy Silences Stat3 Enhances Cartilage Regeneration and Alleviates Osteoarthritis Progression**

Qi Lv, Xiang Zhao, Songsong Teng, Xinmeng Jin, Ying Zhou, Yueyang Sun, Hao Pei,* Zuoqin Yan,* Chunhui Ma,*

**Table S1.** The sequences of oligonucleotides for the triangular DNA origami used in this work

| Name | The sequences (from 5’ to 3’) |
| --- | --- |
| A01 | CGGGGTTTCCTCAAGAGAAGGATTTTGAATTA |
| A02 | AGCGTCATGTCTCTGAATTTACCGACTACCTT |
| A03 | TTCATAATCCCCTTATTAGCGTTTTTCTTACC |
| A04 | ATGGTTTATGTCACAATCAATAGATATTAAAC |
| A05 | TTTGATGATTAAGAGGCTGAGACTTGCTCAGTACCAGGCG |
| A05- Overhangs | TTTGATGATTAAGAGGCTGAGACTTGCTCAGTACCAGGCGTTTTTTTTTTTTTTT |
| A06 | CCGGAACCCAGAATGGAAAGCGCAACATGGCT |
| A07 | AAAGACAACATTTTCGGTCATAGCCAAAATCA |
| A08 | GACGGGAGAATTAACTCGGAATAAGTTTATTTCCAGCGCC |
| A09 | GATAAGTGCCGTCGAGCTGAAACATGAAAGTATACAGGAG |
| A10 | TGTACTGGAAATCCTCATTAAAGCAGAGCCAC |
| A11 | CACCGGAAAGCGCGTTTTCATCGGAAGGGCGA |
| A12 | CATTCAACAAACGCAAAGACACCAGAACACCCTGAACAAA |
| A13 | TTTAACGGTTCGGAACCTATTATTAGGGTTGATATAAGTA |
| A14 | CTCAGAGCATATTCACAAACAAATTAATAAGT |
| A15 | GGAGGGAATTTAGCGTCAGACTGTCCGCCTCC |
| A16 | GTCAGAGGGTAATTGATGGCAACATATAAAAGCGATTGAG |
| A16-Bio | GTCAGAGGGTAATTGATGGCAACATATAAAAGCGATTGAGTTTT-Biotin |
| A17 | TAGCCCGGAATAGGTGAATGCCCCCTGCCTATGGTCAGTG |
| A18 | CCTTGAGTCAGACGATTGGCCTTGCGCCACCC |
| A19 | TCAGAACCCAGAATCAAGTTTGCCGGTAAATA |
| A19-FITC | TCAGAACCCAGAATCAAGTTTGCCGGTAAATATT-FITC |
| A20 | TTGACGGAAATACATACATAAAGGGCGCTAATATCAGAGA |
| A21 | CAGAGCCAGGAGGTTGAGGCAGGTAACAGTGCCCG |
| A22 | ATTAAAGGCCGTAATCAGTAGCGAGCCACCCT |
| A23 | GATAACCCACAAGAATGTTAGCAAACGTAGAAAATTATTC |
| A24 | GCCGCCAGCATTGACACCACCCTC |
| A25 | AGAGCCGCACCATCGATAGCAGCATGAATTAT |
| A25-FITC | AGAGCCGCACCATCGATAGCAGCATGAATTATTT-FITC |
| A26 | CACCGTCACCTTATTACGCAGTATTGAGTTAAGCCCAATA |
| A27 | AGCCATTTAAACGTCACCAATGAACACCAGAACCA |
| A28 | ATAAGAGCAAGAAACATGGCATGATTAAGACTCCGACTTG |
| A29 | CCATTAGCAAGGCCGGGGGAATTA |
| A30 | GAGCCAGCGAATACCCAAAAGAACATGAAATAGCAATAGC |
| A31 | TATCTTACCGAAGCCCAAACGCAATAATAACGAAAATCACCAG |
| A31-Bio | TATCTTACCGAAGCCCAAACGCAATAATAACGAAAATCACCAGTTTT-Biotin |
| A32 | CAGAAGGAAACCGAGGTTTTTAAGAAAAGTAAGCAGATAGCCG |
| A33 | CCTTTTTTCATTTAACAATTTCATAGGATTAG |
| A34 | TTTAACCTATCATAGGTCTGAGAGTTCCAGTA |
| A35 | AGTATAAAATATGCGTTATACAAAGCCATCTT |
| A36 | CAAGTACCTCATTCCAAGAACGGGAAATTCAT |
| A37 | AGAGAATAACATAAAAACAGGGAAGCGCATTA |
| A38 | AAAACAAAATTAATTAAATGGAAACAGTACATTAGTGAAT |
| A39 | TTATCAAACCGGCTTAGGTTGGGTAAGCCTGT |
| A40 | TTAGTATCGCCAACGCTCAACAGTCGGCTGTC |
| A41 | TTTCCTTAGCACTCATCGAGAACAATAGCAGCCTTTACAG |
| A42 | AGAGTCAAAAATCAATATATGTGATGAAACAAACATCAAG |
| A42- Overhangs | AGAGTCAAAAATCAATATATGTGATGAAACAAACATCAAGTTTTTTTTTTTTTTT |
| A43 | ACTAGAAATATATAACTATATGTACGCTGAGA |
| A44 | TCAATAATAGGGCTTAATTGAGAATCATAATT |
| A45 | AACGTCAAAAATGAAAAGCAAGCCGTTTTTATGAAACCAA |
| A45-Bio | AACGTCAAAAATGAAAAGCAAGCCGTTTTTATGAAACCAATTTT-Biotin |
| A46 | GAGCAAAAGAAGATGAGTGAATAACCTTGCTTATAGCTTA |
| A47 | GATTAAGAAATGCTGATGCAAATCAGAATAAA |
| A48 | CACCGGAATCGCCATATTTAACAAAATTTACG |
| A48-FITC | CACCGGAATCGCCATATTTAACAAAATTTACGTT-FITC |
| A49 | AGCATGTATTTCATCGTAGGAATCAAACGATTTTTTGTTT |
| A50 | ACATAGCGCTGTAAATCGTCGCTATTCATTTCAATTACCT |
| A51 | GTTAAATACAATCGCAAGACAAAGCCTTGAAA |
| A52 | CCCATCCTCGCCAACATGTAATTTAATAAGGC |
| A53 | TCCCAATCCAAATAAGATTACCGCGCCCAATAAATAATAT |
| A54 | TCCCTTAGAATAACGCGAGAAAACTTTTACCGACC |
| A55 | GTGTGATAAGGCAGAGGCATTTTCAGTCCTGA |
| A55-FITC | GTGTGATAAGGCAGAGGCATTTTCAGTCCTGATT-FITC |
| A56 | ACAAGAAAGCAAGCAAATCAGATAACAGCCATATTATTTA |
| A57 | GTTTGAAATTCAAATATATTTTAG |
| A58 | AATAGATAGAGCCAGTAATAAGAGATTTAATG |
| A59 | GCCAGTTACAAAATAATAGAAGGCTTATCCGGTTATCAAC |
| A60 | TTCTGACCTAAAATATAAAGTACCGACTGCAGAAC |
| A61 | GCGCCTGTTATTCTAAGAACGCGATTCCAGAGCCTAATTT |
| A62 | TCAGCTAAAAAAGGTAAAGTAATT |
| A63 | ACGCTAACGAGCGTCTGGCGTTTTAGCGAACCCAACATGT |
| A63-Bio | ACGCTAACGAGCGTCTGGCGTTTTAGCGAACCCAACATGTTTTT-Biotin |
| A64 | ACGACAATAAATCCCGACTTGCGGGAGATCCTGAATCTTACCA |
| A65 | TGCTATTTTGCACCCAGCTACAATTTTGTTTTGAAGCCTTAAA |
| B01 | TCATATGTGTAATCGTAAAACTAGTCATTTTC |
| B02 | GTGAGAAAATGTGTAGGTAAAGATACAACTTT |
| B03 | GGCATCAAATTTGGGGCGCGAGCTAGTTAAAG |
| B04 | TTCGAGCTAAGACTTCAAATATCGGGAACGAG |
| B05 | ACAGTCAAAGAGAATCGATGAACGACCCCGGTTGATAATC |
| B05- Overhangs | ACAGTCAAAGAGAATCGATGAACGACCCCGGTTGATAATTTTTTTTTTTTTTTT |
| B06 | ATAGTAGTATGCAATGCCTGAGTAGGCCGGAG |
| B07 | AACCAGACGTTTAGCTATATTTTCTTCTACTA |
| B08 | GAATACCACATTCAACTTAAGAGGAAGCCCGATCAAAGCG |
| B09 | AGAAAAGCCCCAAAAAGAGTCTGGAGCAAACAATCACCAT |
| B10 | CAATATGACCCTCATATATTTTAAAGCATTAA |
| B11 | CATCCAATAAATGGTCAATAACCTCGGAAGCA |
| B12 | AACTCCAAGATTGCATCAAAAAGATAATGCAGATACATAA |
| B13 | CGTTCTAGTCAGGTCATTGCCTGACAGGAAGATTGTATAA |
| B14 | CAGGCAAGATAAAAATTTTTAGAATATTCAAC |
| B15 | GATTAGAGATTAGATACATTTCGCAAATCATA |
| B16 | CGCCAAAAGGAATTACAGTCAGAAGCAAAGCGCAGGTCAG |
| B16-Bio | CGCCAAAAGGAATTACAGTCAGAAGCAAAGCGCAGGTCAGTTTT-Biotin |
| B17 | GCAAATATTTAAATTGAGATCTACAAAGGCTACTGATAAA |
| B18 | TTAATGCCTTATTTCAACGCAAGGGCAAAGAA |
| B19 | TTAGCAAATAGATTTAGTTTGACCAGTACCTT |
| B19-FITC | TTAGCAAATAGATTTAGTTTGACCAGTACCTTTT-FITC |
| B20 | TAATTGCTTTACCCTGACTATTATGAGGCATAGTAAGAGC |
| B21 | ATAAAGCCTTTGCGGGAGAAGCCTGGAGAGGGTAG |
| B22 | TAAGAGGTCAATTCTGCGAACGAGATTAAGCA |
| B23 | AACACTATCATAACCCATCAAAAATCAGGTCTCCTTTTGA |
| B24 | ATGACCCTGTAATACTTCAGAGCA |
| B25 | TAAAGCTATATAACAGTTGATTCCCATTTTTG |
| B25-FITC | TAAAGCTATATAACAGTTGATTCCCATTTTTGTT-FITC |
| B26 | CGGATGGCACGAGAATGACCATAATCGTTTACCAGACGAC |
| B27 | TAATTGCTTGGAAGTTTCATTCCAAATCGGTTGTA |
| B28 | GATAAAAACCAAAATATTAAACAGTTCAGAAATTAGAGCT |
| B29 | ACTAAAGTACGGTGTCGAATATAA |
| B30 | TGCTGTAGATCCCCCTCAAATGCTGCGAGAGGCTTTTGCA |
| B31 | AAAGAAGTTTTGCCAGCATAAATATTCATTGACTCAACATGTT |
| B31-Bio | AAAGAAGTTTTGCCAGCATAAATATTCATTGACTCAACATGTTTTTT-Biotin |
| B32 | AATACTGCGGAATCGTAGGGGGTAATAGTAAAATGTTTAGACT |
| B33 | AGGGATAGCTCAGAGCCACCACCCCATGTCAA |
| B34 | CAACAGTTTATGGGATTTTGCTAATCAAAAGG |
| B35 | GCCGCTTTGCTGAGGCTTGCAGGGGAAAAGGT |
| B36 | GCGCAGACTCCATGTTACTTAGCCCGTTTTAA |
| B37 | ACAGGTAGAAAGATTCATCAGTTGAGATTTAG |
| B38 | CCTCAGAACCGCCACCCAAGCCCAATAGGAACGTAAATGA |
| B39 | ATTTTCTGTCAGCGGAGTGAGAATACCGATAT |
| B40 | ATTCGGTCTGCGGGATCGTCACCCGAAATCCG |
| B41 | CGACCTGCGGTCAATCATAAGGGAACGGAACAACATTATT |
| B42 | AGACGTTACCATGTACCGTAACACCCCTCAGAACCGCCAC |
| B42- Overhangs | AGACGTTACCATGTACCGTAACACCCCTCAGAACCGCCACTTTTTTTTTTTTTTT |
| B43 | CACGCATAAGAAAGGAACAACTAAGTCTTTCC |
| B44 | ATTGTGTCTCAGCAGCGAAAGACACCATCGCC |
| B45 | TTAATAAAACGAACTAACCGAACTGACCAACTCCTGATAA |
| B45-Bio | TTAATAAAACGAACTAACCGAACTGACCAACTCCTGATAATTTT-Biotin |
| B46 | AGGTTTAGTACCGCCATGAGTTTCGTCACCAGGATCTAAA |
| B47 | GTTTTGTCAGGAATTGCGAATAATCCGACAAT |
| B48 | GACAACAAGCATCGGAACGAGGGTGAGATTTG |
| B48-FITC | GACAACAAGCATCGGAACGAGGGTGAGATTTGTT-FITC |
| B49 | TATCATCGTTGAAAGAGGACAGATGGAAGAAAAATCTACG |
| B50 | AGCGTAACTACAAACTACAACGCCTATCACCGTACTCAGG |
| B51 | TAGTTGCGAATTTTTTCACGTTGATCATAGTT |
| B52 | GTACAACGAGCAACGGCTACAGAGGATACCGA |
| B53 | ACCAGTCAGGACGTTGGAACGGTGTACAGACCGAAACAAA |
| B54 | ACAGACAGCCCAAATCTCCAAAAAAAAATTTCTTA |
| B55 | AACAGCTTGCTTTGAGGACTAAAGCGATTATA |
| B55-FITC | AACAGCTTGCTTTGAGGACTAAAGCGATTATATT-FITC |
| B56 | CCAAGCGCAGGCGCATAGGCTGGCAGAACTGGCTCATTAT |
| B57 | CGAGGTGAGGCTCCAAAAGGAGCC |
| B58 | ACCCCCAGACTTTTTCATGAGGAACTTGCTTT |
| B59 | ACCTTATGCGATTTTATGACCTTCATCAAGAGCATCTTTG |
| B60 | CGGTTTATCAGGTTTCCATTAAACGGGAATACACT |
| B61 | AAAACACTTAATCTTGACAAGAACTTAATCATTGTGAATT |
| B62 | GGCAAAAGTAAAATACGTAATGCC |
| B63 | TGGTTTAATTTCAACTCGGATATTCATTACCCACGAAAGA |
| B63-Bio | TGGTTTAATTTCAACTCGGATATTCATTACCCACGAAAGATTTT-Biotin |
| B64 | ACCAACCTAAAAAATCAACGTAACAAATAAATTGGGCTTGAGA |
| B65 | CCTGACGAGAAACACCAGAACGAGTAGGCTGCTCATTCAGTGA |
| C01 | TCGGGAGATATACAGTAACAGTACAAATAATT |
| C02 | CCTGATTAAAGGAGCGGAATTATCTCGGCCTC |
| C03 | GCAAATCACCTCAATCAATATCTGCAGGTCGA |
| C04 | CGACCAGTACATTGGCAGATTCACCTGATTGC |
| C05 | TGGCAATTTTTAACGTCAGATGAAAACAATAACGGATTCG |
| C05- Overhangs | TGGCAATTTTTAACGTCAGATGAAAACAATAACGGATTCGTTTTTTTTTTTTTTT |
| C06 | AAGGAATTACAAAGAAACCACCAGTCAGATGA |
| C07 | GGACATTCACCTCAAATATCAAACACAGTTGA |
| C08 | TTGACGAGCACGTATACTGAAATGGATTATTTAATAAAAG |
| C09 | CCTGATTGCTTTGAATTGCGTAGATTTTCAGGCATCAATA |
| C10 | TAATCCTGATTATCATTTTGCGGAGAGGAAGG |
| C11 | TTATCTAAAGCATCACCTTGCTGATGGCCAAC |
| C12 | AGAGATAGTTTGACGCTCAATCGTACGTGCTTTCCTCGTT |
| C13 | GATTATACACAGAAATAAAGAAATACCAAGTTACAAAATC |
| C14 | TAGGAGCATAAAAGTTTGAGTAACATTGTTTG |
| C15 | TGACCTGACAAATGAAAAATCTAAAATATCTT |
| C16 | AGAATCAGAGCGGGAGATGGAAATACCTACATAACCCTTC |
| C16-Bio | AGAATCAGAGCGGGAGATGGAAATACCTACATAACCCTTCTTTT-Biotin |
| C17 | GCGCAGAGGCGAATTAATTATTTGCACGTAAATTCTGAAT |
| C18 | AATGGAAGCGAACGTTATTAATTTCTAACAAC |
| C19 | TAATAGATCGCTGAGAGCCAGCAGAAGCGTAA |
| C19-FITC | TAATAGATCGCTGAGAGCCAGCAGAAGCGTAATT-FITC |
| C20 | GAATACGTAACAGGAAAAACGCTCCTAAACAGGAGGCCGA |
| C21 | TCAATAGATATTAAATCCTTTGCCGGTTAGAACCT |
| C22 | CAATATTTGCCTGCAACAGTGCCATAGAGCCG |
| C23 | TTAAAGGGATTTTAGATACCGCCAGCCATTGCGGCACAGA |
| C24 | ACAATTCGACAACTCGTAATACAT |
| C25 | TTGAGGATGGTCAGTATTAACACCTTGAATGG |
| C25-FITC | TTGAGGATGGTCAGTATTAACACCTTGAATGGTT-FITC |
| C26 | CTATTAGTATATCCAGAACAATATCAGGAACGGTACGCCA |
| C27 | CGCGAACTAAAACAGAGGTGAGGCTTAGAAGTATT |
| C28 | GAATCCTGAGAAGTGTATCGGCCTTGCTGGTACTTTAATG |
| C29 | ACCACCAGCAGAAGATGATAGCCC |
| C30 | TAAAACATTAGAAGAACTCAAACTTTTTATAATCAGTGAG |
| C31 | GCCACCGAGTAAAAGAACATCACTTGCCTGAGCGCCATTAAAA |
| C31-Bio | GCCACCGAGTAAAAGAACATCACTTGCCTGAGCGCCATTAAAATTTT-Biotin |
| C32 | TCTTTGATTAGTAATAGTCTGTCCATCACGCAAATTAACCGTT |
| C33 | CGCGTCTGATAGGAACGCCATCAACTTTTACA |
| C34 | AGGAAGATGGGGACGACGACAGTAATCATATT |
| C35 | CTCTAGAGCAAGCTTGCATGCCTGGTCAGTTG |
| C36 | CCTTCACCGTGAGACGGGCAACAGCAGTCACA |
| C37 | CGAGAAAGGAAGGGAAGCGTACTATGGTTGCT |
| C38 | GCTCATTTTTTAACCAGCCTTCCTGTAGCCAGGCATCTGC |
| C39 | CAGTTTGACGCACTCCAGCCAGCTAAACGACG |
| C40 | GCCAGTGCGATCCCCGGGTACCGAGTTTTTCT |
| C41 | TTTCACCAGCCTGGCCCTGAGAGAAAGCCGGCGAACGTGG |
| C42 | GTAACCGTCTTTCATCAACATTAAAATTTTTGTTAAATCA |
| C42- Overhangs | GTAACCGTCTTTCATCAACATTAAAATTTTTGTTAAATCATTTTTTTTTTTTTTTT |
| C43 | ACGTTGTATTCCGGCACCGCTTCTGGCGCATC |
| C44 | CCAGGGTGGCTCGAATTCGTAATCCAGTCACG |
| C45 | TAGAGCTTGACGGGGAGTTGCAGCAAGCGGTCATTGGGCG |
| C45-Bio | TAGAGCTTGACGGGGAGTTGCAGCAAGCGGTCATTGGGCGTTTT-Biotin |
| C46 | GTTAAAATTCGCATTAATGTGAGCGAGTAACACACGTTGG |
| C47 | TGTAGATGGGTGCCGGAAACCAGGAACGCCAG |
| C48 | GGTTTTCCATGGTCATAGCTGTTTGAGAGGCG |
| C48-FITC | GGTTTTCCATGGTCATAGCTGTTTGAGAGGCGTT-FITC |
| C49 | GTTTGCGTCACGCTGGTTTGCCCCAAGGGAGCCCCCGATT |
| C50 | GGATAGGTACCCGTCGGATTCTCCTAAACGTTAATATTTT |
| C51 | AGTTGGGTCAAAGCGCCATTCGCCCCGTAATG |
| C52 | CGCGCGGGCCTGTGTGAAATTGTTGGCGATTA |
| C53 | CTAAATCGGAACCCTAAGCAGGCGAAAATCCTTCGGCCAA |
| C54 | CGGCGGATTGAATTCAGGCTGCGCAACGGGGGATG |
| C55 | TGCTGCAAATCCGCTCACAATTCCCAGCTGCA |
| C55-FITC | TGCTGCAAATCCGCTCACAATTCCCAGCTGCATT-FITC |
| C56 | TTAATGAAGTTTGATGGTGGTTCCGAGGTGCCGTAAAGCA |
| C57 | TGGCGAAATGTTGGGAAGGGCGAT |
| C58 | TGTCGTGCACACAACATACGAGCCACGCCAGC |
| C59 | CAAGTTTTTTGGGGTCGAAATCGGCAAAATCCGGGAAACC |
| C60 | TCTTCGCTATTGGAAGCATAAAGTGTATGCCCGCT |
| C61 | TTCCAGTCCTTATAAATCAAAAGAGAACCATCACCCAAAT |
| C62 | GCGCTCACAAGCCTGGGGTGCCTA |
| C63 | CGATGGCCCACTACGTATAGCCCGAGATAGGGATTGCGTT |
| C63-Bio | CGATGGCCCACTACGTATAGCCCGAGATAGGGATTGCGTTTTTT-Biotin |
| C64 | CGATGGCCCACTACGTATAGCCCGAGATAGGGATTGCGTT |
| C65 | ACGTGGACTCCAACGTCAAAGGGCGAATTTGGAACAAGAGTCC |
| Link-A1C | TTAATTAATTTTTTACCATATCAAA |
| Link-A2C | TTAATTTCATCTTAGACTTTACAA |
| Link-A3C | CTGTCCAGACGTATACCGAACGA |
| Link-A4C | TCAAGATTAGTGTAGCAATACT |
| Link-B1A | TGTAGCATTCCTTTTATAAACAGTT |
| Link-B2A | TTTAATTGTATTTCCACCAGAGCC |
| Link-B3A | ACTACGAAGGCTTAGCACCATTA |
| Link-B4A | ATAAGGCTTGCAACAAAGTTAC |
| Link-C1B | GTGGGAACAAATTTCTATTTTTGAG |
| Link-C2B | CGGTGCGGGCCTTCCAAAAACATT |
| Link-C3B | ATGAGTGAGCTTTTAAATATGCA |
| Link-C4B | ACTATTAAAGAGGATAGCGTCC |
| Loop | GCGCTTAATGCGCCGCTACAGGGC |
| si-Stat3-F | GACUUUGAUUUCAACUAUA |
| si-Stat3-R | UAUAGUUGAAAUCAAAGUCAAAAAAAAAA |
| Bio-CD44 | Biotin-ACCGGGCGTACACCGTCGCGGCACATGTCTGAATGCGTTTAGTCTCTGTG |


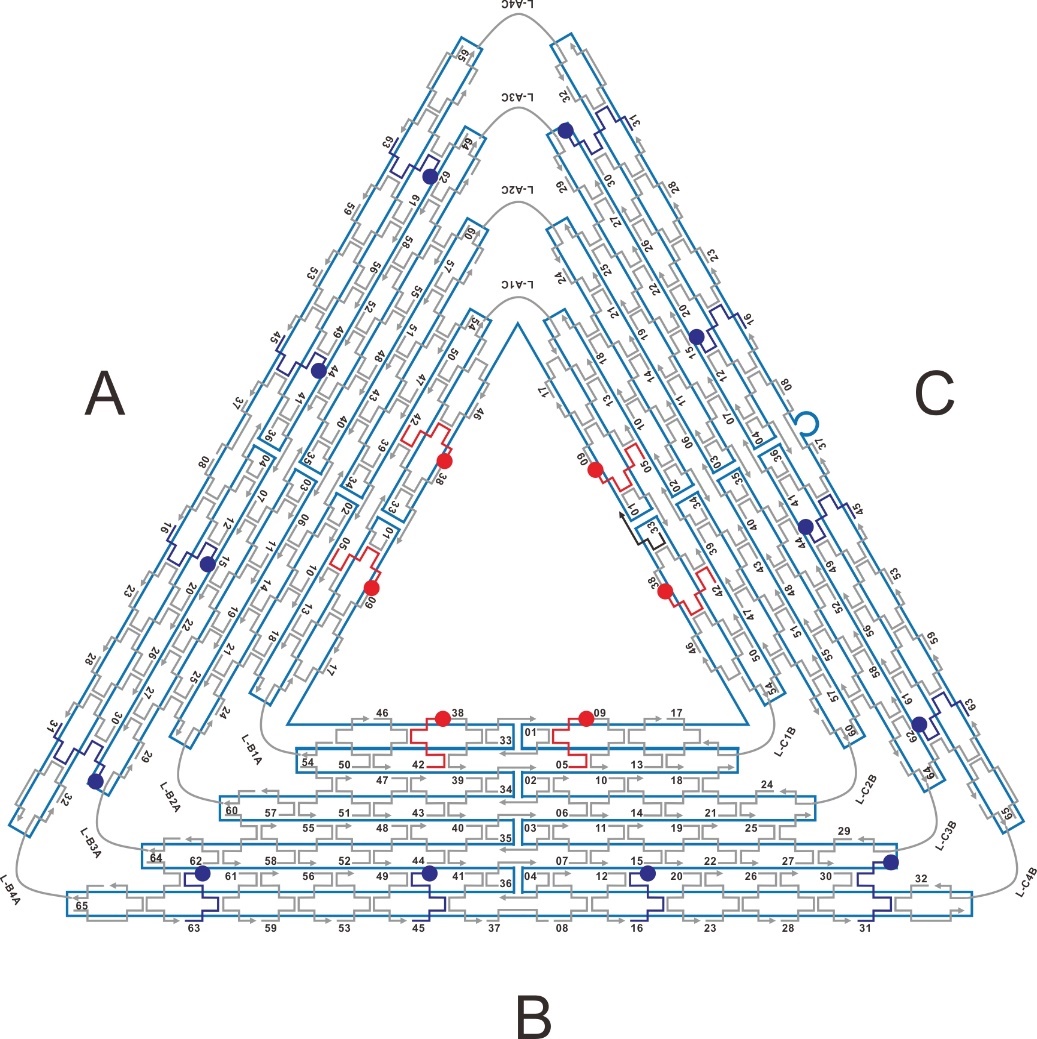


**Figure S1.** Scheme design of triangle DNA origami. The M13mp18 scaffold is shown in blue, staple strands are in gray. Red dots indicate overhang positions for hybridization with complementary si-Stat3 sequences. Blue dots mark biotinylated sites for anti-CD44 aptamer attachment. (Sequences are listed in Table S1).


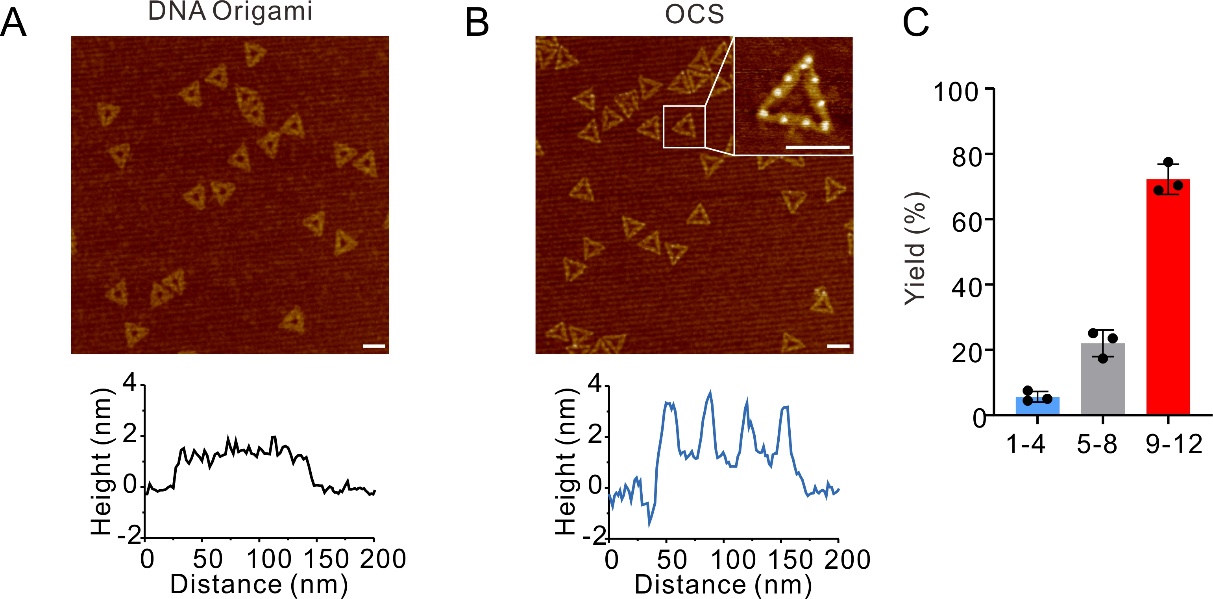


**Figure S2.** A) AFM image of the functional triangular DNA origami scaffolds. Measure the cross-sectional height of the functional triangular DNA origami scaffolds. Scale bar: 100 nm. B) AFM images of the functional triangular DNA origami scaffolds loaded with si-Stat3 and anti-CD44 aptamer, respectively. The bright spots in the images represent anti-CD44 aptamers anchored via streptavidin-biotin interactions. Measure the cross-sectional height of OCS. Scale bar: 100 nm. C) Histogram showing the number of visible highlight spots per DNA origami from AFM images, corresponding to the expected twelve binding sites.


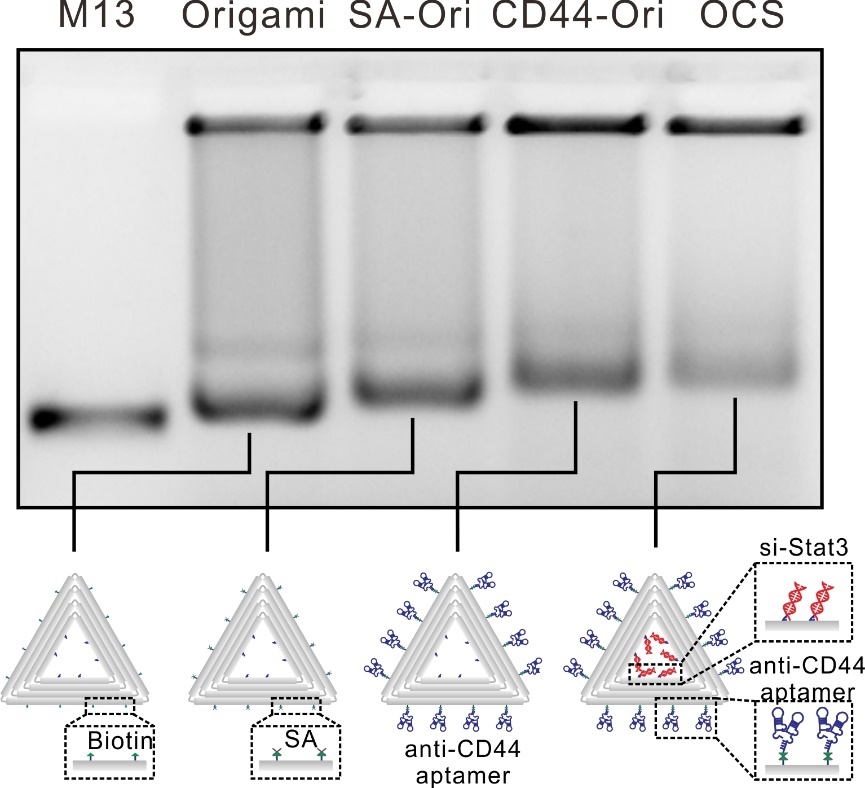


**Figure S3.** Analysis of the successful binding of si-Stat3 and anti-CD44 aptamers by agarose gel electrophoresis (1%). From left to right: M13mp18 scaffold, triangular DNA origami, SA modified triangular DNA origami, anti-CD44 aptamer modified triangular DNA origami, anti-CD44 aptamer and si-Stat3 modified triangular DNA origami.


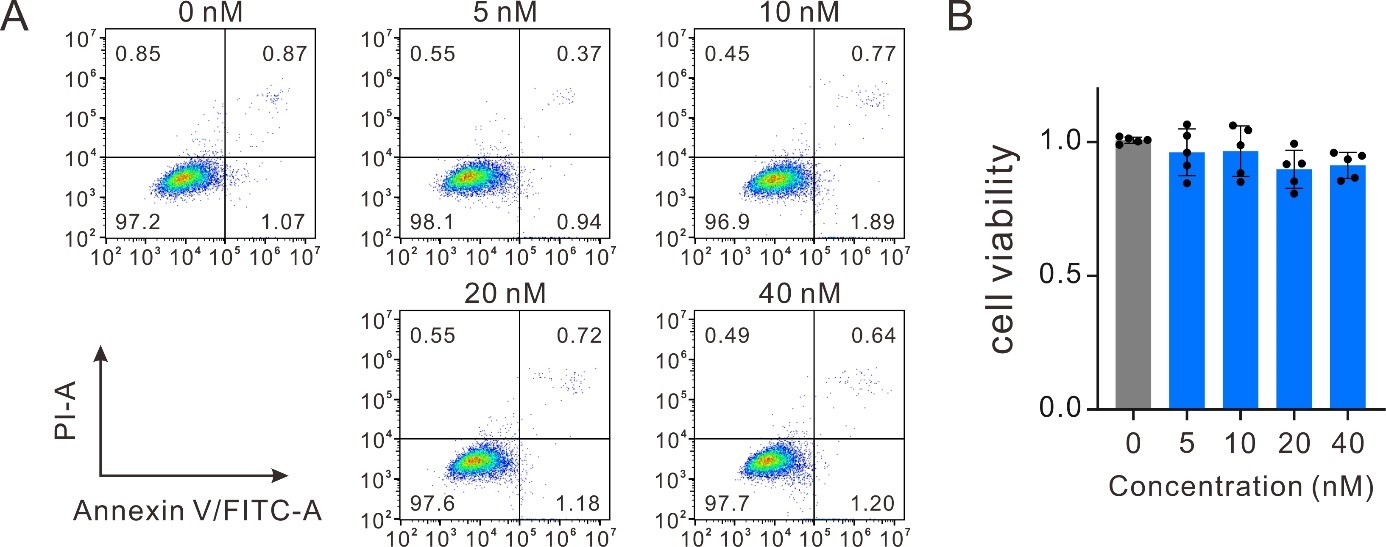


**Figure S4.** A) Flow cytometric analysis of apoptosis in chondrocytes incubated with different concentrations of OCS for 12 hours. B) MTT assay for assessing cell viability in chondrocytes incubated with different concentrations of OCS for 24 hours. Data were presented as means ± SD (n=5).


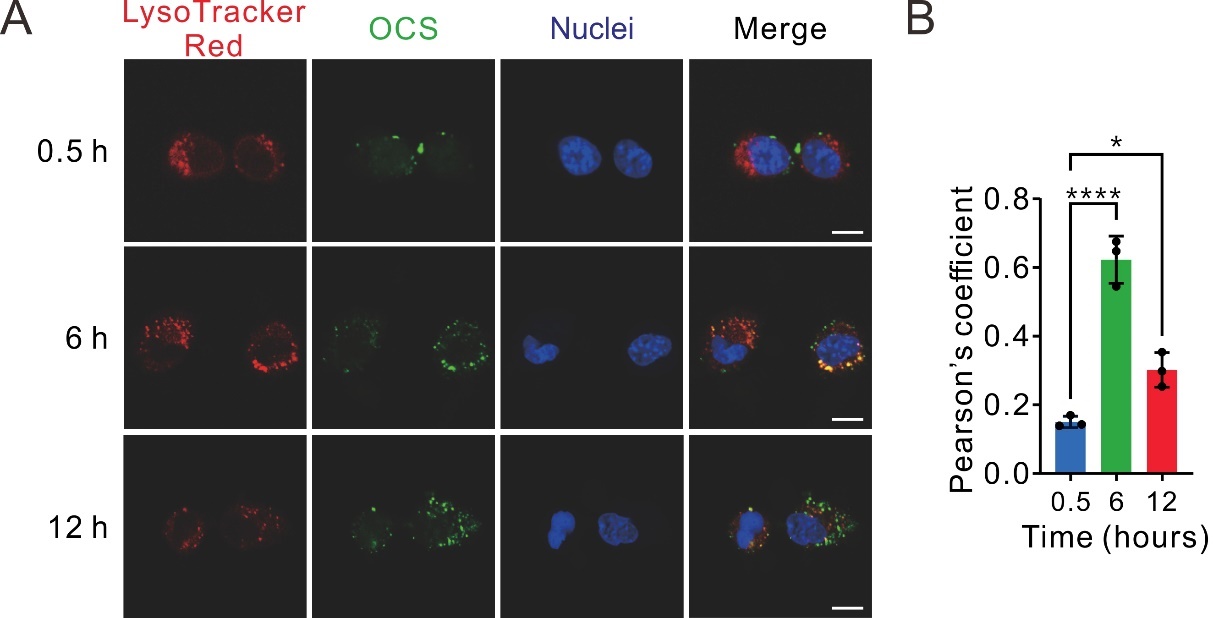


**Figure S5.** A) Endosomal escape analysis of OCS in IL-1β-induced diseased chondrocytes. Endosomes are labeled with LysoTracker Red, OCS are labeled with FITC, and nuclei are labeled with DAPI. Scale bar: 10 μm. B) Quantification of lysosomal escape using Pearson’s correlation coefficient. Data were presented as mean ± SD (n = 3). Statistical comparisons were performed using one-way ANOVA. **p*<0.05, ****p*<0.001, ns indicates no statistical significance.


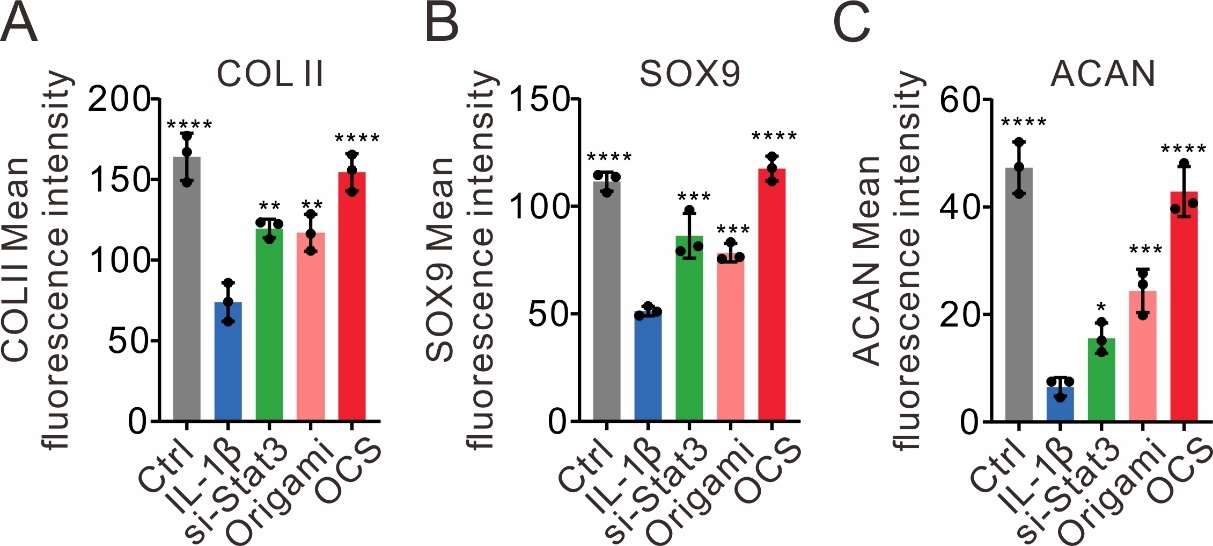


**Figure S6.** Relative fluorescence intensity analysis of COL-II (A), SOX9 (B) and ACAN (C) in relevant Figure 3H-J. Data were presented as means ± SD (n=3). Statistical comparisons were performed using one-way ANOVA. **p*<0.05, ***p*<0.01, ****p*<0.001, *****p*<0.0001.


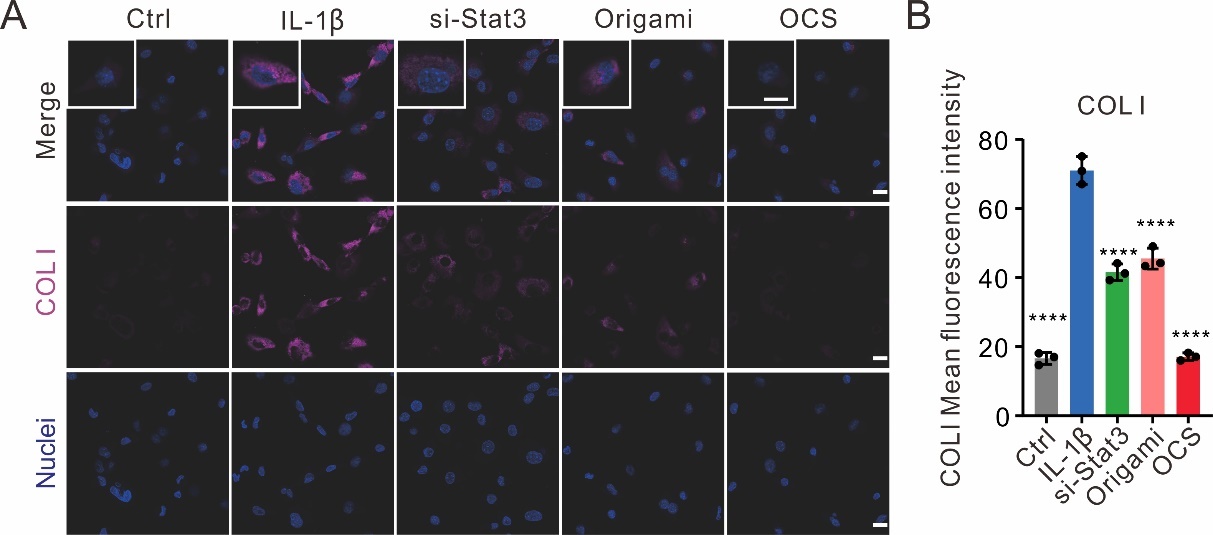


**Figure S7.** A) Observation of protein expression of COL-I in chondrocytes under different treatment conditions by immunofluorescence. Scale bar: 20 μm. B) Relative fluorescence intensity analysis of COL-I. Data were presented as means ± SD (n=3). Statistical comparisons were performed using one-way ANOVA. *****p*<0.0001.


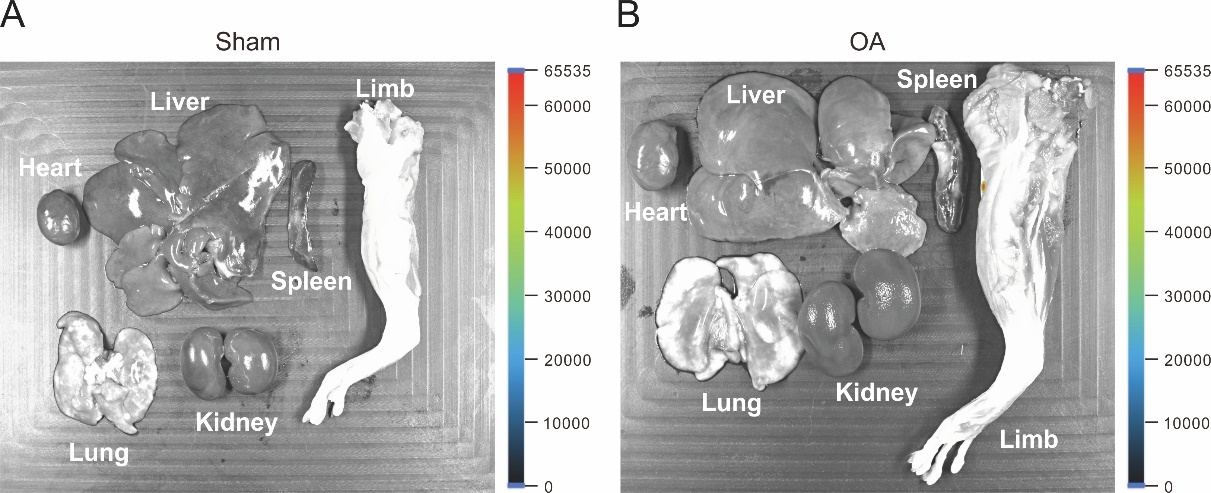


**Figure S8.** A-B) Representative ex vivo fluorescence images of major organs and knee joints obtained from Sham and OA groups after 120 hours of rat knee joint injection.


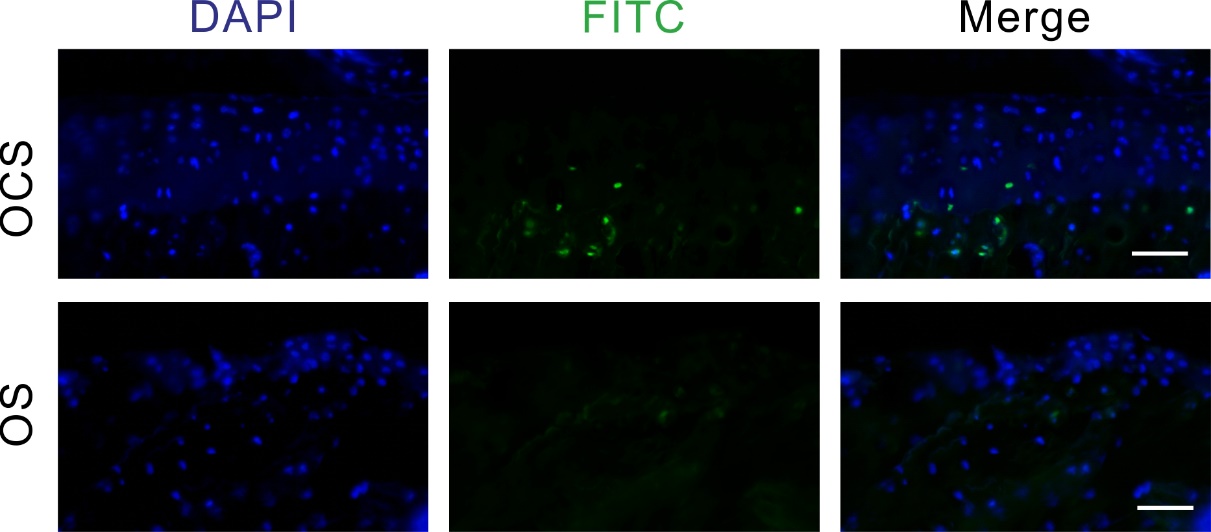


**Figure S9.** CD44-dependent targeting assessment in vivo. OA rats were intra-articularly injected with FITC-labeled OCS (containing anti-CD44 aptamers) or control FITC-OC (lacking anti-CD44 aptamers). Twenty-four hours post-injection, knee joints were harvested, frozen sections prepared, and cell nuclei stained with DAPI.


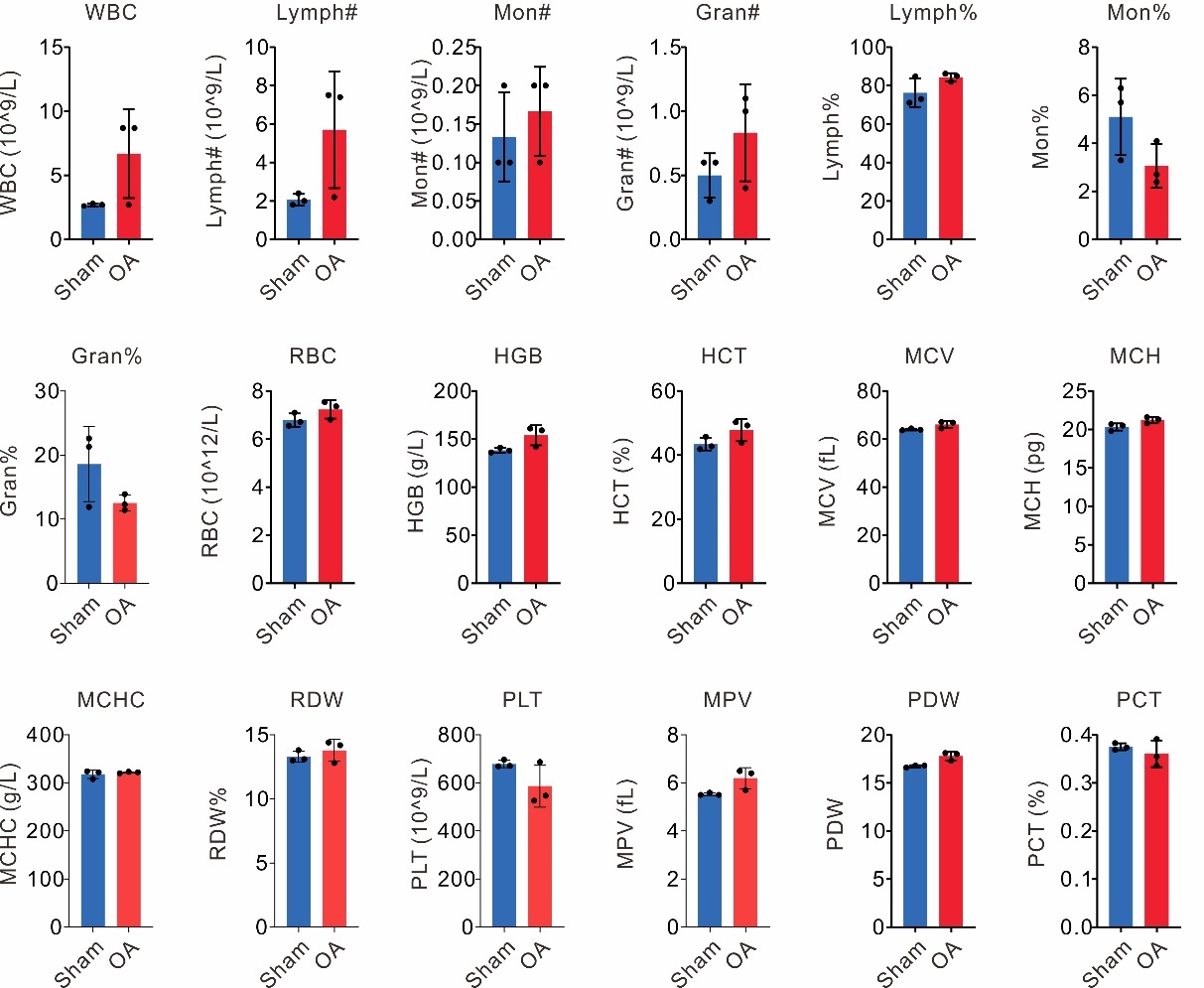


**Figure S10.** Blood routine analysis of Sham and OA groups. Data were presented as means ± SD (n=3).


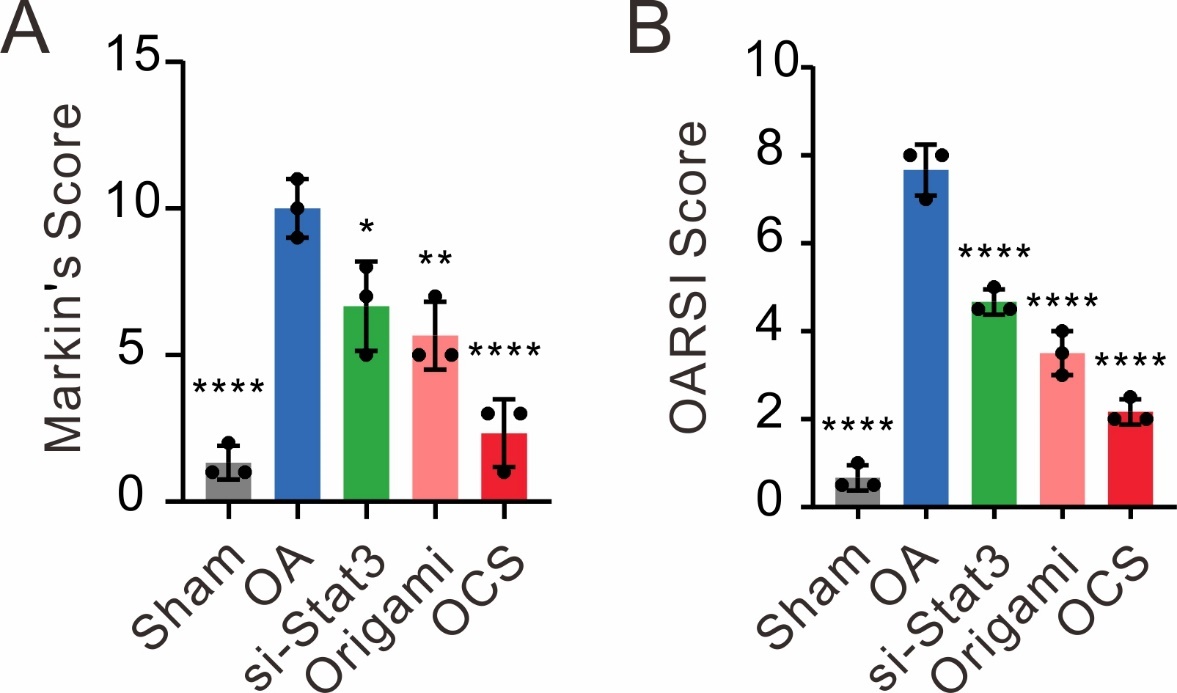


**Figure S11.** A) Mankin's scoring system for histological assessment of cartilage degeneration in osteoarthritis. Data are shown as the mean ± S.D (n=3). B) OARSI scoring system for the histological assessment of the severity of osteoarthritis. Data were presented as means ± SD (n=3). Statistical comparisons were performed using one-way ANOVA. **p*<0.05, ***p*<0.01, ****p*<0.001, *****p*<0.0001.


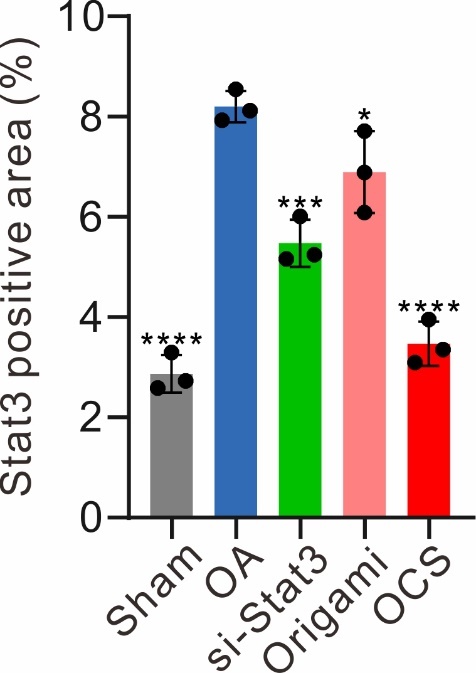


**Figure S12.** The Stat3-positive (Stat3^+^) area calculated from Figure 6E. Data were presented as means ± SD (n=3). Statistical comparisons were performed using one-way ANOVA. **p*<0.05, ***p*<0.01, ****p*<0.001, *****p*<0.0001.
